# Metabolic and lipidomic investigation of the antiproliferative effects of coronatine against human melanoma cells

**DOI:** 10.1038/s41598-019-39990-w

**Published:** 2019-02-28

**Authors:** Hye-Youn Kim, Hanyong Jin, Jeehyeon Bae, Hyung-Kyoon Choi

**Affiliations:** College of Pharmacy, Chung-Ang University, Seoul, 06974 Republic of Korea

## Abstract

Melanoma is the most aggressive form of skin cancer, with metastatic melanoma being refractory to currently available conventional therapies. In this study, we evaluated the inhibitory effect of coronatine (COR) on the proliferation of metastatic melanoma cells. COR inhibited the proliferation of melanoma cells but negligibly affected the proliferation of normal melanocytes. Comparative metabolic and lipidomic profiling using gas chromatography-mass spectrometry and direct infusion-mass spectrometry was performed to investigate COR-induced metabolic changes. These analyses identified 33 metabolites and 82 lipids. Of these, the levels of lactic acid and glutamic acid, which are involved in energy metabolism, significantly decreased in COR-treated melanoma cells. Lipidomic profiling indicated that ceramide levels increased in COR-treated melanoma cells, suggesting that ceramides could function as a suppressor of cancer cell proliferation. In contrast, the levels of phosphatidylinositol (PI) species, including PI 16:0/18:0, 16:0/18:1, 18:0/18:0, and 18:0/18:1, which were found to be potential biomarkers of melanoma metastasis in our previous study, were lower in the COR-treated cells than in control cells. The findings of metabolomic and lipidomic profiling performed in the present study provide new insights on the anticancer mechanisms of COR and can be used to apply COR in cancer treatment.

## Introduction

Malignant melanoma is a highly aggressive type of skin cancer that metastasizes to almost any internal organ; moreover, the incidence of melanoma has increased steadily over the past three decades^1^. Although malignant melanoma accounts for only 4% of all cutaneous malignancies, it is responsible for the majority of skin cancer-related deaths^2^. The global cancer statistics indicates that malignant melanoma is the third most commonly diagnosed cancer in Australia (Melanoma Institute Australia; http://www.melanoma.org.au) and the fifth most commonly diagnosed cancer in the United States^1^. If diagnosed at an early stage, melanoma can be easily treated through surgical resection. However, management of metastatic melanoma is challenging because of the unavailability of drugs that reliably affect its disease course. The lack of effective treatment options for patients with metastatic melanoma is mainly attributed to the resistance of this cancer to conventional chemotherapeutic agents^3^. Therefore, novel compounds and therapeutic strategies are needed to control melanoma metastasis.

Approximately 25% of the currently used anticancer drugs are directly derived from plants; moreover, recent studies have highlighted the enormous potential of many phytohormones as anticancer agents^4–9^. In plants, plant hormones play a pivotal role in regulating defensive responses to invading pathogens by triggering programmed cell death (PCD) near the infection site^10^. Recent studies have suggested that mechanisms associated with the modulation of PCD execution are similar in both plants and animals. Moreover, several lines of evidence suggest that some PCD regulators are conserved between plant and mammalian cells. The transgenic expression of anti- or proapoptotic proteins (Bcl-xL, Ced-9, p35, or Bax) affects the suppression or activation of cell death in plants cells similar to that in animal cells. In tobacco plants, Bcl-xL and Ced-9 overexpression inhibits cell death induced by ultraviolet B (UVB) irradiation (32 kJ/m^2^), herbicide treatment, or tobacco mosaic virus infection. Transgenic tomato plants expressing *p35* show protection against mycotoxin-induced cell death and pathogen infection. In contrast, expression of murine *Bax* activates cell death in tobacco plants^11–13^. In mammalian cells, plant hormones such as abscisic acid, salicylic acid, and jasmonic acid regulate PCD and exhibit anticancer activities both *in vitro* and *in vivo*^6,14,15^. Particularly, methyl jasmonate (MJ), a plant stress hormone belonging to jasmonic acid family, induces cell death in and impairs the metastasis of various cancers such as breast, lung, and gastric cancers and melanoma^15–18^. One study showed that MJ inhibited the migration of murine metastatic melanoma B16-F10 cells and highly metastatic B16 COL/R melanoma cells that exhibited multidrug resistance and significantly suppressed the metastasis of melanoma to the lungs of C57BL mice^15^.

Coronatine (COR), a phytotoxin produced by the pathovars of plant bacteria *Pseudomonas syringae*, functions as a growth regulator and stimulator of secondary metabolite biosynthesis in plants^19–21^. COR, a structural analog of MJ, consists of coronafacic acid and coronamic acid derived from isoleucine; it has been suggested to mimic the action of MJ^19^. In a previous study, COR and MJ exerted similar effects on the inhibition of root growth, stimulation of anthocyanin accumulation, and upregulation of jasmonate-induced ~29- and ~31-kD proteins in *Arabidopsis*; moreover, COR-insensitive *Arabidopsis* mutants were insensitive to MJ^20^. The structural similarity between MJ and COR and the analogy between their biological responses in plants led us to hypothesize that COR can regulate cell death in cancer cells similar to MJ.

Cancer metabolism has emerged as a major theme in cancer research, and metabolomic and lipidomic studies have provided comprehensive information on tumor progression and have improved our understanding of mechanisms underlying cancer pathogenesis and drug effects^22–26^. Recent studies have identified biomarkers and therapeutic targets in lung, breast, ovarian, and colon cancers and melanoma using analytical techniques in metabolomics and have evaluated the effects of therapeutic compounds by assessing key metabolic changes in colorectal cancer and melanoma^27–34^.

Most cancer cells show high glycolysis levels because of the production of energy and nutrients needed for their proliferation; therefore, glycolysis inhibition is a promising strategy for anticancer therapy^35,36^. 2-Deoxy-d-glucose (2-DG), a synthetic glucose analog, competitively inhibits glucose uptake; moreover, phosphorylated 2-DG (2-DG-6-phosphate) cannot be metabolized further, leading to ATP depletion and oxidative stress^37–39^. Although treatment with 2-DG alone does not significantly induce cell death, treatment with a combination of 2-DG with specific agents or radiation exerts synergistic anticancer effects^40^. One study showed that 2-DG increased cisplatin- and staurosporine-induced apoptotic rates in human metastatic melanoma cell lines (MeWo and Mel-501)^41^. In addition, combined treatment with MJ and 2-DG enhanced ATP depletion and cell death in lung, colon, and breast cancer cell lines (D122, CT26, and MCF7), and 2-DG treatment attenuated the resistance of the sarcoma cell line MCA-105 to MJ, thereby sensitizing these cells to MJ-induced cytotoxicity^42,43^. However, no study has investigated the effects of COR alone or in combination with 2-DG on human melanoma cells to date. The present study mainly assessed the synergistic anticancer effects of COR and 2-DG on human metastatic melanoma cell lines A375 and A2058 and examined cellular metabolic and lipidomic alterations induced by these compounds in the melanoma cells. The response of cancer cells to COR and 2-DG combination treatment was determined using metabolomics and lipidomics approaches by performing gas chromatography-mass spectrometry (GC-MS) and direct infusion-mass spectrometry (DI-MS) combined with multivariate statistical analysis.

## Results

### MJ or COR inhibits the proliferation of human melanoma cells

**Figure 1:**
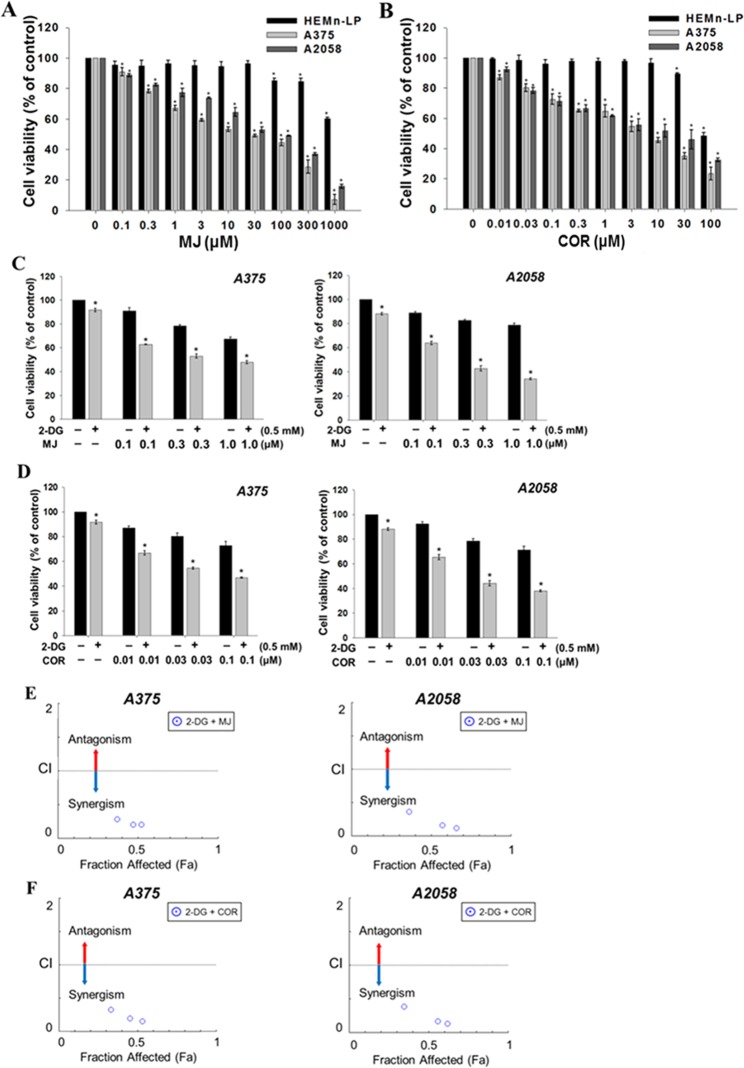
Growth inhibitory effect of MJ or COR on the human melanocytes (HEMn-LP) and melanoma cells (A375 and A2058) with low and high metastatic potentials. The cells were treated with (**A**) MJ (0–1000 μM) or (**B**) COR (0–100 μM) for 24 h, and cell viability was assessed by performing the MTT assay. Combination treatment with (**C**) MJ (0.1, 0.3, or 1.0 μM) or (**D**) COR (0.01, 0.03, or 0.1 μM) with 2-DG (0.5 mM) exerted synergistic cytotoxic effects on the melanoma cells. CI plots for treatment with the combinations (**E**) 2-DG + MJ and (**F**) 2-DG + COR were generated to evaluate the effect of the two compounds in combination with 2-DG. The bars indicate mean values, and the error bars indicate the standard deviation of triplicate experiments. The asterisk (*) denotes significant difference compared with the control group, as determined using the Student’s *t*-test (*p* < 0.05). CI, combination index; COR, coronatine; 2-DG, 2-Deoxy-d-glucose; MJ, methyl jasmonate; MTT, 3-(4,5-dimethylthiazol-2-yl)−2,5-diphenyltetrazolium bromide.

The effect of MJ and COR on the growth of melanoma cells or normal melanocytes was investigated by assessing the viability of HEMn-LP, A375, and A2058 cells treated with different doses of MJ and COR for 24 h by performing 3-(4,5-dimethylthiazol-2-yl)−2,5-diphenyltetrazolium bromide (MTT) assay. MJ or COR treatment decreased the viability of melanoma A375 and A2058 cells in a dose-dependent but negligibly affected the viability of normal human epidermal melanocytes HEMn-LP (Fig. 1A,B). The median inhibitory concentration (IC_50_) values of MJ and COR were 13.18 ± 1.50 and 4.60 ± 0.51 μM, respectively, for A375 cells and 37.94 ± 1.18 and 7.93 ± 1.50 μM, respectively, for A2058 (Table 1).

**Table 1:** The IC_50_ values of MJ and COR alone and CI values of the combination of MJ or COR with 2-DG (0.5 mM) against melanoma cells.

Cell line	MJ		COR	
	IC_50_ (μM)	CI	IC_50_ (μM)	CI
A375	13.18 ± 1.50	0.29	4.60 ± 0.51	0.33
A2058	37.94 ± 1.18	0.36	7.93 ± 1.50	0.39

The IC_50_ and CI values were calculated using the CompuSyn software. The CI values were estimated from the combination effect data of 0.1 μM MJ + 0.5 mM 2-DG and 0.01 μM COR + 0.5 mM 2-DG. CI value of <1, 1, or >1 indicates synergistic, additive, or antagonistic effect, respectively. Data are expressed as mean ± SD of triplicate experiments. CI, combination index; COR, coronatine; 2-DG, 2-Deoxy-d-glucose; MJ, methyl jasmonate.

### The combination of MJ or COR with 2-DG exerts synergistic antiproliferative effects on human melanoma cells

**Figure 2:**
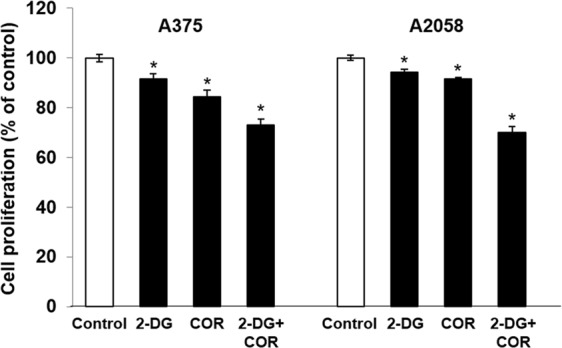
The effect of the combination of 2-DG and COR on the proliferation of the human melanoma cell lines. The A375 and A2058 cells were treated with 0.5 mM 2-DG and 0.01 μM COR, and BrdU cell proliferation assay were performed at 24 h after the treatment. The bars indicate mean values, and the error bars indicate the standard error of the mean of three independent experiments (n = 6). The asterisk (*) denotes significant difference compared with the control group, as determined using the Student’s *t*-test (*p* < 0.05).

Previous studies have shown that 2-DG-induced glycolysis inhibition enhances the effect of MJ on lung, colon, and breast cancer and sarcoma cells^42,43^. Therefore, we determined the effects of the combination of MJ and 2-DG and of COR and 2-DG on melanoma cells. MJ and COR concentrations were chosen such that cell viability was maintained at >60% in accordance with dose-response data (Fig. 1C,D). The synergistic effects of the combination treatments were determined by calculating combination index (CI) values, which depict synergism quantitatively. For both A375 and A2058 cells, the CI values of the combination treatment with 0.5 mM 2-DG and MJ or COR ranged from 0.12 to 0.39 (Fig. 1E,F). The combination of 2-DG with MJ and of 2-DG with COR exerted strong synergistic effects, as indicated by CI values of <1. Because a lower concentration of COR than that of MJ exerted synergistic antiproliferative effects along with 2-DG on the melanoma cells in the present study, we selected the combination treatment with 0.01 μM COR and 0.5 mM 2-DG for performing metabolomics and lipidomics analyses. At this concentration, COR negligibly affected the viability of normal melanocytes after 96 h of exposure (Supplementary Fig. S1). To validate the antiproliferative effect of COR on melanoma cells, we examined the changes in A375 and A2058 cell proliferation after treatment with the combination of COR and 2-DG by performing 5′-bromo-2′-deoxyuridine (BrdU) incorporation enzyme-linked immunosorbent assay (ELISA). The percentage of melanoma cells showing the incorporation of BrdU, a marker of replicative DNA synthesis in S-phase cells, significantly decreased after treatment with the combination of COR and 2-DG (Fig. 2), which was consistent with the results of the MTT assay.

### Treatment with the combination of COR and 2-DG alters the relative abundance of metabolites and lipids in melanoma cells

The metabolic and lipidomic changes induced by COR alone or in combination with 2-DG were investigated using the human melanoma A375 and A2058 cells, which have different metastatic potentials, and primary HEMn-LP melanocytes by performing the GC-MS and DI-MS analyses. The GC-MS analysis detected the following 33 metabolites: two alcohols, fifteen amino acids, four organic acids, three purines, two pyrimidines, five sugars, and two other metabolites (Supplementary Table S1). The relative changes in metabolite levels are shown as fold changes, which are log_2_ ratios of normalized intensities of the identified metabolites in treated cells compared with those in control cells (Supplementary Table S2). Notably, treatment with 1.0 μM COR alone or with the combination of 0.01 μM COR and 0.5 mM 2-DG induced similar changes in the levels of organic acids; sugars; and amino acids, including alanine, aspartic acid, glutamic acid, proline, and threonine, in the two melanoma cell lines. The levels of these metabolites significantly decreased after COR treatment. Particularly, the levels of sugars showed a higher decrease in A375 cells treated with the combination of COR with 2-DG than in those treated with COR alone. The levels of metabolites involved in energy metabolism, such as glucose, glucose-6-phosphate, lactic acid, fumaric acid, and malic acid, were lower in the melanoma cells treated with COR alone and with COR + 2-DG than in control cells. However, the levels of the glycolytic metabolites, including glucose, glucose-6-phosphate, and lactic acid, were not significantly altered and the levels of fumaric acid and malic acid were increased in COR-treated normal melanocytes. Moreover, COR treatment decreased the levels of purines and pyrimidines such as hypoxanthine, inosine, guanine, uracil, and uridine in A375 cells. However, no significant differences were noted in the levels of purines between the treated and control A2058 cells. Significant changes in metabolite levels were also observed in the normal melanocytes; however, these changes were lower than those observed in the melanoma cells.

**Figure 3:**
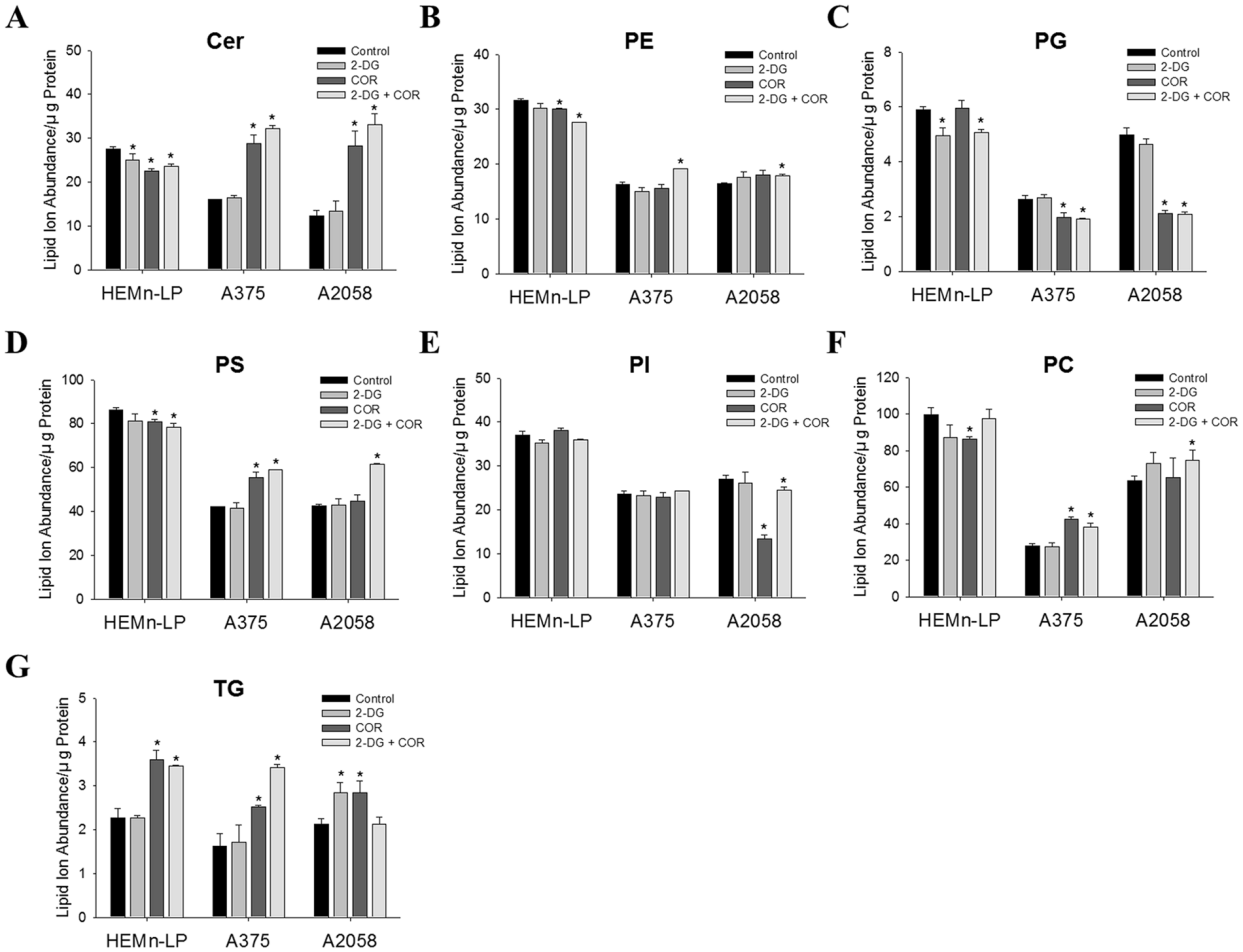
Comparison of the total summed relative abundance of each lipid class between the control and treatment (2-DG, COR, and 2-DG + COR) groups on human melanocytes (HEMn-LP) and melanoma cells (A375 and A2058). The relative abundance of lipids in the cells was quantified by comparing the intensities of lipid ion peaks with the intensity of the internal standard and subsequent normalization of quantified ion abundances with protein equivalents used for cellular lipid profiling. Each bar on the graph represents the sum of the protein-adjusted normalized lipid abundance for all the identified lipid species within each lipid class, and the error bars indicate the standard deviation of triplicate experiments. The asterisk (*) indicates significant differences compared with the control group, as determined using Student’s *t*-test (*p* < 0.05). (**A**) Cer, ceramide; (**B**) PE, phosphatidylethanolamine; (**C**) PG, phosphatidylglycerol; (**D**) PS, phosphatidylserine; (**E**) PI, phosphatidylinositol; (**F**) PC, phosphatidylcholine; and (**G**) TG, triacylglycerol.

Various lipid molecules regulate cellular processes, including cell growth and proliferation, membrane homeostasis, metastasis, apoptosis, and drug response^44^. COR-induced alterations in lipid metabolism were investigated by performing lipidomic profiling with nano-electrospray ionization-mass spectrometry (nanoESI-MS). In all, 17 lipid species, including 11 phosphatidylcholine (PC) and 6 triacylglycerol (TG) species, were detected in the positive ion mode and 65 lipid species, including 9 ceramide (Cer), 15 phosphatidylethanolamine (PE), 3 phosphatidylglycerol (PG), 25 phosphatidylserine (PS), and 13 phosphatidylinositol (PI) species, were detected in the negative ion mode. Detailed information on the proposed compositions of fatty acyl chins, ion species, mass-to-charge ratio (*m/z*) values, MS/MS fragment ion, and fold changes (log_2_ ratio) of intact lipid species in the control and treated cells is listed in Supplementary Tables S3 and S4. The total relative abundance of each lipid class in the melanocytes and melanoma cells was determined as the sum of individual lipid species of that class (Fig. 3). The total levels of Cer, PS, PC, and TG species were higher in the A375 cells in the COR and 2-DG + COR treated groups than in those in the control group. Moreover, the total Cer, PS, and PC levels increased in the the A2058 cells in the 2-DG + COR treatment group. In contrast, the total PI level significantly decreased only in the A2058 cells in both the treatment groups.

All the identified Cer species accumulated in both A375 and A2058 melanoma cells treated with COR alone and with the combination of COR and 2-DG. Particularly, Cer (d18:1/18:0) showed the highest accumulation compared with other fatty acyl chain-containing species. In contrast, the levels of all PG species (PG 16:0/18:1, PG 18:1/18:1, and PG 18:0/18:1) were lower in the COR-treated cells than in the control cells, with PG 18:1/18:1 level showing a marked decrease in the COR-treated cells. Our previous study showed that the levels of PI species, including PI 16:0/18:0, PI 16:0/18:1, PI 18:0/18:0, and PI 18:0/18:1, increased progressively in melanoma cells with higher metastatic potential and suggested that these PI species were potential novel biomarkers of melanoma metastasis^45^. Interestingly, in the present study, treatment with COR alone decreased the levels of these PI species in the two melanoma cell lines compared with those in the control cells. Treatment with the combination of COR and 2-DG decreased the levels of PI 16:0/18:1 and PI 18:0/18:1 but did not affect the levels of PI 16:0/18:0 and PI 18:0/18:0. In normal melanocyte, COR treatment decreased the levels of most Cer species. In addition, no significant differences were observed in the levels of most PI species between the COR-treated and control cells.

### Multivariate statistical analysis of the metabolic and lipidomic profiles of melanoma cells treated with the combination of COR and 2-DG

**Figure 4:**
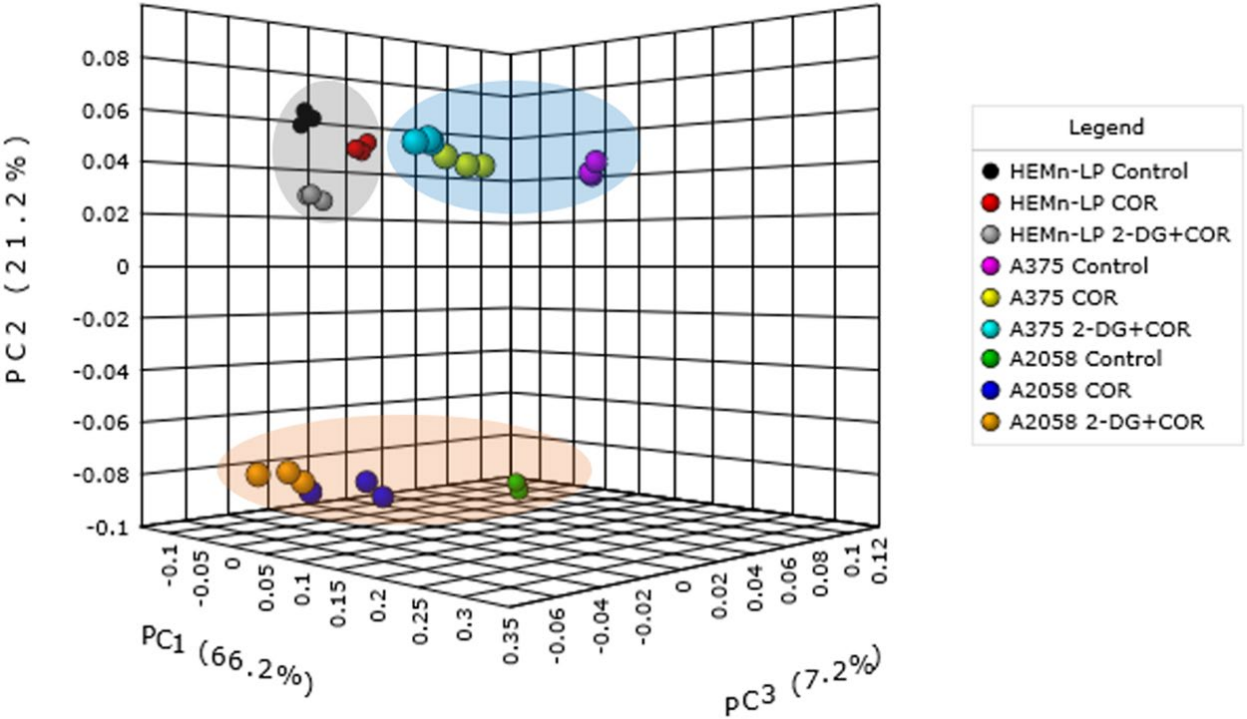
PCA score plot derived from the metabolite and lipid profiling data of the normal melanocytes (HEMn-LP) and melanoma cell lines (A375 and A2058) in the control and treatment (COR or 2-DG + COR) groups.

The COR-induced changes in the metabolite and lipid profiles of the normal melanocytes and two melanoma cell lines were compared by analyzing the GC-MS and DI-MS spectral datasets through principle component analysis (PCA) (Fig. 4). In score plots, the first three principal components accounted for 94.6% total variance and reflected most of the spectral information. The two melanoma groups showed obvious separation from the normal group. Moreover, a clear separation was observed between the two melanoma groups. Interestingly, the control samples and the samples treated with COR alone or with the combination of COR and 2-DG were closer to one another in the normal group. However, the COR and COR + 2-DG treated samples were remote from the untreated control samples in the melanoma groups. These results implied that the COR and 2-DG combination treatment induced significant perturbations in the metabolite and lipid profiles of the melanoma cells.

**Figure 5:**
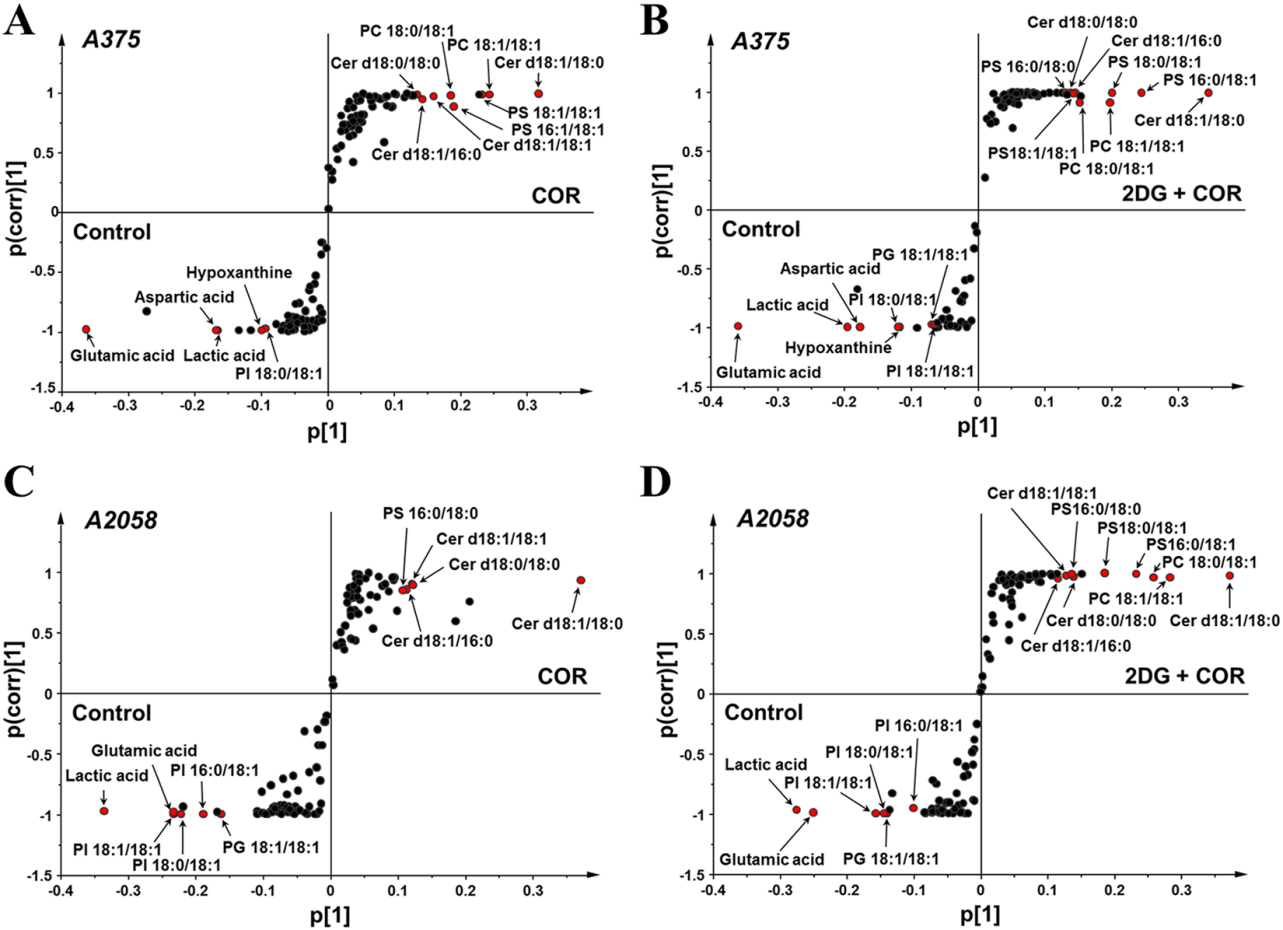
The S-plots derived from metabolite and lipid profiling data of (**A**) A375 cells in the control vs. COR treatment groups, (**B**) A375 cells in the control vs. 2-DG + COR treatment groups, (**C**) A2058 cells in the control vs. COR treatment groups, and (**D**) A2058 cells in the control vs. 2-DG + COR treatment groups.

**Figure 6:**
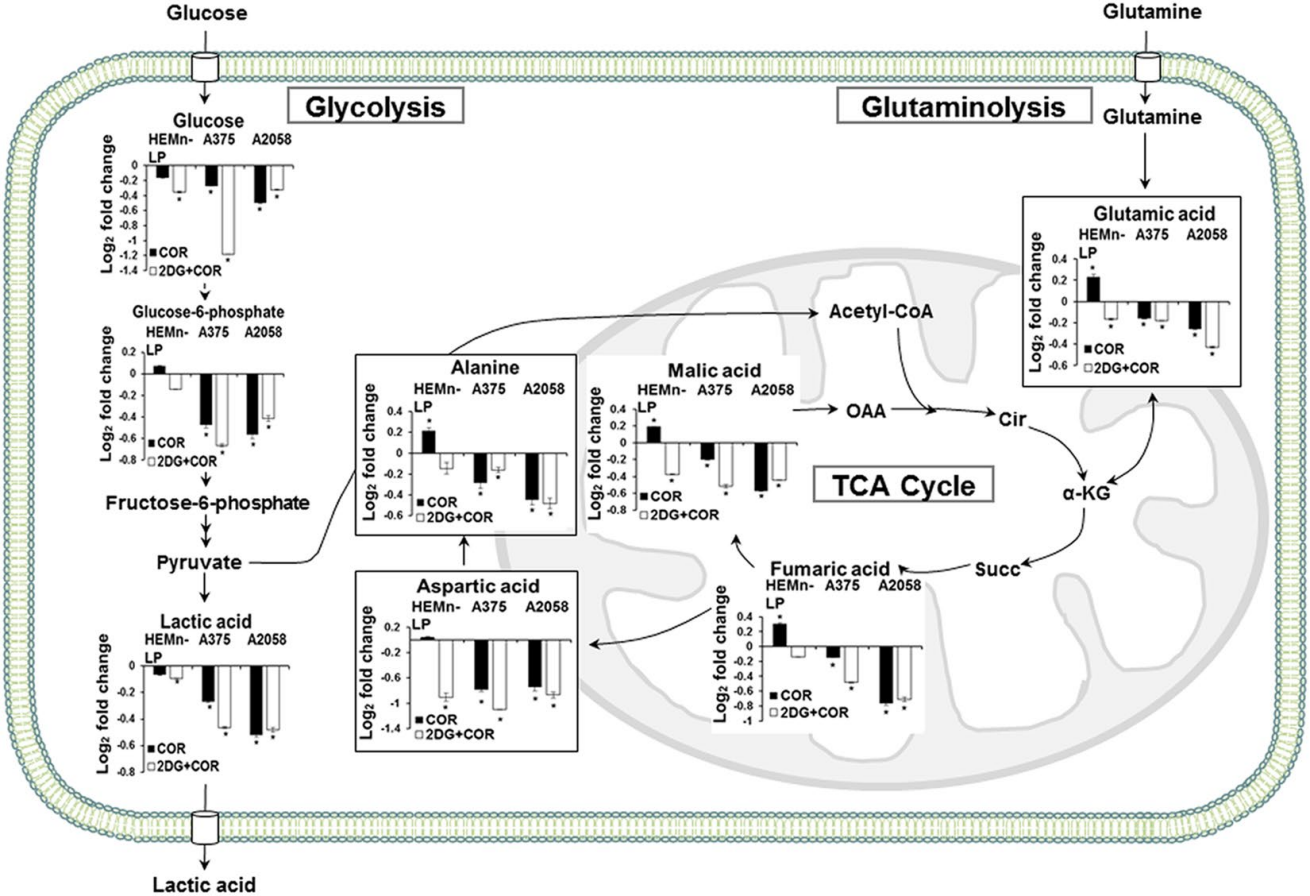
Alteration of energy metabolism in the melanoma cells treated with COR alone or with the combination of COR and 2-DG. Fold changes are shown relative to those in the control cells, and the error bars represent the standard deviation of triplicate experiments. The asterisk (*) denotes statistically significant differences compared with the control group, as determined using Student’s *t*-test (*p* < 0.05).

**Figure 7:**
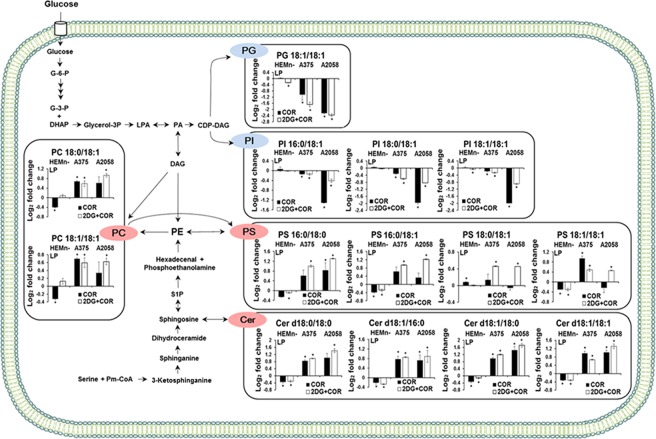
Alterations in glycerophospholipid and sphingolipid metabolism in melanoma cells induced by treatment with COR alone or combined treatment with 2-DG. Fold changes in lipid species strongly contributing to group separation based on the S-plots are shown relative to those in the control cells. The error bars represent the standard deviation of triplicate experiments. The asterisk (*) denotes statistically significant differences compared with the control group, as determined using the Student’s *t*-test (*p* < 0.05). The proposed pathways were based on data available in the KEGG database (http://www.genome.jp/kegg/). CDP-DAG, cytidine diphosphate-diacylglycerol; Cer, ceramide; DAG, diacylglycerol; LPA, lysophosphatidic acid; PA, phosphatidic acid; PC, phosphatidylcholine; PE, phosphatidylethanolamine; PG, phosphatidylglycerol; PI, phosphatidylinositol; Pm-CoA, palmitoyl coenzyme A; PS, phosphatidylserine; S1P, sphingosine-1-phosphate.

Next, the metabolites and lipids that showed maximum changes after COR treatment were identified by obtaining S-plots by comparing untreated groups with the COR or COR + 2-DG treated groups in A375 and A2058 melanoma cells through orthogonal projection to latent structure-discriminant analysis (OPLS-DA). Model quality is indicated by parameters *R*^2^*Y* and *Q*^2^*Y*, which represent the goodness of fit and predictability of the model, respectively. Validity was assessed using the analysis of variance of cross-validated predictive residuals; *p* < 0.05^46^. The parameters for each model are listed in Supplementary Table S5. The S-plots combine a scatter plot p[1], which describes the magnitude of each variable within a model, and p(corr)[1], which represents the reliability of each variable (modeled correlation). The most significantly altered variables are plotted in the lower left or upper right of the S-plot and are highly correlated with group separation^47^. In the present study, 10 compounds at the outermost bottom and top of the S-plots were selected as potentially relevant metabolites altered by COR treatment based on a p(corr) cutoff value of |0.8|. Candidate predictive markers of response to COR treatment were selected from these compounds based on the principle VIP value of >1.0 and on compounds whose levels commonly increased or decreased in more than two groups treated with COR (Supplementary Table S6). These compounds are highlighted using red filled circles in Fig. 5. The lower left region in the figure shows the compounds whose levels decreased after COR treatment. These compounds include glutamic acid, lactic acid, aspartic acid, hypoxanthine, three PI species (PI 16:0/18:1, PI 18:0/18:1, and PI 18:1/18:1), and PG 18:1/18:1. The upper right region in the figure shows the compounds whose levels increased after COR treatment. These compounds include four Cer species (Cer d18:1/18:0, Cer d18:1/18:1, Cer d18:1/16:0, and Cer d18:0/18:0), four PS species (PS 16:0/18:0, PS 16:0/18:1, PS 18:0/18:1, and PS 18:1/18:1), and two PC species (PC 18:0/18:1 and PC 18:1/18:1). Fragmentation mass spectra of these compounds are shown in Supplementary Fig. S2. Among these selected candidate markers, glutamic acid, lactic acid, Cer d18:1/18:0, Cer d18:1/16:0, and Cer d18:0/18:0 showed the same variation tendency in all the COR treated groups. Therefore, these compounds were indicated as potential biomarkers for evaluating the therapeutic effect of COR on melanoma cells. The main altered metabolic and lipidomic pathways in the COR treated melanoma cells are shown in Figs 6 and 7. These pathways included glycerophospholipid and sphingolipid metabolism, and energy metabolism such as glycolysis, TCA cycle, and glutaminolysis.

In the same way, S-plot derived from OPLS-DA was used to identify the major compounds changed in response to COR treatment in normal melanocytes, and these compounds are highlighted using red filled circles (Supplementary Fig. S3). The decrease in serine, plasmenyl-PE 18:0/22:5, two Cer species (Cer d18:1/18:0, Cer d18:1/18:1), and four PS species (PS 14:0/16:0, PS 16:0/18:1, PS 16:1/18:1, PS 18:1/18:1) levels was the major metabolic and lipidomic change in the melanocytes treated with COR alone and with COR + 2-DG. The levels of Cer d18:1/18:0, Cer d18:1/18:1, PS 16:0/18:1, and PS 18:1/18:1 were decreased after COR treatment in melanocytes, whereas these levels were increased after COR treatment in melanoma cells. The decrease in these lipids could associated with protective mechanism against COR-induced cell death in normal melanocytes.

## Discussion

The metabolic status of a cell is closely associated with its growth, survival, and proliferation. In this study, we investigated the effect of COR and 2-DG on the normal melanocytes and two metastatic melanoma cell lines by performing comprehensive metabolic and lipidomic profiling and identified major metabolic alterations induced by COR treatment. First, we found that COR was more effective than MJ, a structural COR analog that exerts anticancer effects on metastatic melanoma cells, in inhibiting the growth of melanoma cells and exerted negligible effects on the growth of normal cells^15^. Treatment with the combination of COR and 2-DG significantly decreased the levels glycolysis and TCA cycle metabolites in the melanoma cells. Most cancer cells highly depend on glycolysis to meet their increased energy and biosynthesis demands for proliferation and differentiation. Therefore, COR treatment-induced decrease in glycolysis in melanoma cells may be effective for inhibiting the growth of and inducing cell death in cancer cells^35,48–50^. Inhibition of glucose metabolism markedly decreases ATP levels in cancer cells and sensitizes myeloid leukemia and cervical, breast, and prostate cancer cells to death receptor-induced apoptosis^51,52^. In the present study, the COR and 2-DG combination treatment exerted an enhanced inhibitory effect on the growth of melanoma cells. 2-DG reduces glycolytic enzyme activity and decreases intracellular ATP level^53–55^. A previous study showed that treatment with the combination of 2-DG with cisplatin and staurosporine, which are proapoptotic drugs, increased cell death in human metastatic melanoma cells by inducing apoptosis^41^.

In the present study, decrease in lactic and glutamic acid levels was the most relevant COR-induced metabolic alteration in the metastatic melanoma cells. Because lactic acid accumulation through glycolysis induces acidosis in cells, tumor cells promote lactic acid efflux through monocarboxylate transporters to maintain pH homeostasis^56,57^. This reduces extracellular pH and enhances the migration and invasion of human melanoma cells^58–60^. Therefore, the COR-induced decrease in lactic acid levels observed in the present study suggests the inhibition of glucose metabolism in and suppression of the invasion and metastasis of melanoma cells. The initial step of glutaminolysis, a major metabolic pathway contributing to tumor growth, is the conversion of glutamine to glutamic acid, which is an important source for biosynthesis pathways and energy in proliferating cancer cells^61,62^. Glutamic acid, a precursor of α-ketoglutarate, serves as a carbon source to replenish the TCA cycle and is degraded to citrate, pyruvate, aspartic acid, alanine, serine, and proline to provide metabolic intermediates for cell maintenance^63,64^. Thus, the decreased glutamic acid level in the COR-treated cells relative to that in the control cells may reflect metabolic changes that suppress the growth of melanoma cells. Moreover, the decreased alanine and aspartate levels in the COR-treated cells may be closely associated with disturbances in alanine, aspartate, and glutamate metabolism, which is the most relevant pathway in melanoma development and had the highest impact score in pathway analysis performed in our previous study^45^.

The melanoma cells treated with COR showed significant accumulation of various Cer species, particularly Cer with an 18-carbon chain, such as Cer d18:1/18:1, Cer d18:1/18:0, and Cer d18:0/18:0. Cer, a powerful tumor suppressor, limits cancer cell proliferation by inducing apoptosis, cell cycle arrest, and autophagic cell death. Moreover, Cer forms Cer-enriched membrane platforms that cluster death receptors such as TNF-related apoptosis-inducing ligand receptor and CD95 to activate apoptotic signaling pathways^65,66^. Thus, the role of Cer in cell death suggests that Cer metabolism is an attractive therapeutic target. Several studies have examined Cer-based therapies involving agents that increase Cer level for cancer treatment. For example, taxol induces cellular Cer generation in breast cancer cells (MCF-7 and MDA-MB-468), thus increasing their apoptosis^67^. In addition, treatment with 1-phenyl-2-decanoylamino-3-morpholino-1-propanol, a Cer glucosylation inhibitor, results in the concomitant accumulation of Cer and enhances curcumin-induced apoptosis of B16 and WM-115 melanoma cells^68^. Exogenously added cell-permeable short-chain Cer (C6) exerts cytotoxic effects on human melanoma cell lines^69,70^. Therefore, elevation of intracellular Cer species in the COR-treated melanoma cells may be important for suppressing the proliferation of these cells.

In the present study, COR treatment significantly altered the levels of glycerophospholipids, which are major lipid components of biological membranes, and inhibited the growth of melanoma cells. The COR-treated cells showed increased levels of TG species, which was consistent with that reported previously in various cell types in response to apoptotic stimuli. Early TG accumulation after Fas mAb treatment is associated with apoptosis onset in Jurkat T-cells. Shotgun lipidomics profiling showed that TG species were accumulated in apoptotic keratinocytes treated with narrow-band UVB irradiation^71,72^. Although the exact role of TG accumulation is unknown at present, an increase in fatty acid level after TG accumulation is suggested to contribute to Cer generation, which eventually induces cell death^73^. The levels of most PS species increased in the melanoma cells treated with the combination of COR and 2-DG. This increase in the levels of PS species may be associated with altered PS metabolism during apoptosis. PS externalization to the outer plasma membrane is an early apoptotic marker, and PS biosynthesis is stimulated in different apoptotic cells^74^. In camptothecin-induced apoptotic human leukemia U937 cells, the total synthesis of PS is stimulated greater than other major membrane phospholipids such as PC, PE, and sphingomyelin. Jurkat cells undergoing CD95-induced apoptosis show enhanced PS synthesis by inhibiting the formation of PE through decarboxylation of the PS. Particularly, newly synthesized PS is preferentially translocated to the outer leaflet of the plasma membrane; thus, increased PS synthesis may contribute to the phagocytosis of apoptotic cells^75,76^. In the PI class, the levels of PI species with saturated and monounsaturated fatty acyl chains decreased in the COR-treated melanoma cells. Interestingly, the levels of PI 16:0/18:0, PI 16:0/18:1, PI 18:0/18:0, and PI 18:0/18:1, whose accumulation is suggested to be a potential biomarker of melanoma metastasis in our previous study, decreased after COR treatment^45^. PI and its metabolites play an important role in various cellular responses. Particularly, phosphatidylinositol-3-kinase (PI3K)/Akt pathway is critical for melanoma initiation and therefore is a therapeutic target^77–79^. Previous studies showed that PI3K inhibitors prevented Akt activation, induced apoptosis of various melanoma cell lines, and inhibited tumor growth in an *in vivo* mouse model of melanoma brain metastasis^78,80,81^. Although the association between the levels of PI and inhibition of cancer cell proliferation remains unclear, inhibitors of PI synthesis suppress the growth of cancer cells, including small cell lung carcinoma and oral squamous carcinoma cells^82,83^. Together, these findings suggest that the COR treatment-induced reduction in the levels of signaling lipids such as PI phosphates and PI suppresses the growth and proliferation of melanoma cells by inactivating the PI3K/Akt pathway.

Thus, the present study investigated the effects of COR on the proliferation of metastatic melanoma cells by performing metabolite and lipid profiling combined with multivariate statistical analysis. To our knowledge, this is the first study to show the metabolic and lipidomic alterations in COR-treated melanoma cells. The results of the present study showed that COR treatment suppressed the growth of melanoma cells by inhibiting glycolysis, TCA cycle, and glutaminolysis, which play essential roles in energy production during cancer cell proliferation. In addition, COR treatment induced the accumulation of Cer, a tumor suppressor, and increased the levels of TG and PS. Interestingly, the levels of PI species, including PI 16:0/18:0, PI 16:0/18:1, PI 18:0/18:0, and PI 18:0/18:1, which are suggested to be the potential biomarkers of melanoma metastasis, decreased in the COR-treated melanoma cells. This decrease in the levels of PI species was suggested to inhibit cancer cell growth. Together, these results provide new insights on the major metabolic and lipidomic alterations induced by COR in melanoma cells and offer a basis for using COR in melanoma treatment. Future *in vivo* studies assessing the toxicity, antiproliferative activity, and mechanisms under the antiproliferative activity of COR will help in determining its potential for treating metastatic melanoma.

## Methods

### Chemicals and reagents

High-performance liquid chromatography-grade chloroform, methanol, and water were purchased from Fisher Scientific (Pittsburg, PA). Ammonium acetate, butylated hydroxytoluene, COR, 2-DG, dimethyl sulfoxide (DMSO), MJ, methoxyamine hydrochloride, myristic-d_27_ acid, and pyridine were purchased from Sigma-Aldrich (St. Louis, MO). *N*,*O*-Bis(trimethylsilyl)trifluoroacetamide (BSTFA) containing 1% trimethylchlorosilane (TMCS) was purchased from Alfa Aesar (Ward Hill, MA), and 1,2-diheptadecanoyl-sn-glycero-3-phosphoethanolamine was purchased from Avanti Polar Lipids (Alabaster, AL).

### Cell culture

The primary human epidermal melanocytes HEMn-LP were cultured in medium 254 supplemented with human melanocyte growth supplement (Cascade Biologics, Portland, OR). The human metastatic melanoma cell lines A375 and A2058 (American Type Culture Collection, Manassas, VA) were maintained in Dulbecco’s modified Eagle’s medium supplemented with 10% fetal bovine serum and 1% penicillin-streptomycin (Hyclone Labs, Logan, UT). All the cells were cultured at 37 °C in a humidified incubator with an atmosphere of 5% CO_2_ and were subcultured when they reached 90% confluency.

### Cell viability and combination treatment analysis

The cells were seeded in 96-well microtiter plates at a density of 2 × 10^4^ cells/well and were incubated at 37 °C for 24 h. Next, the cells were treated with MJ, COR, or 2-DG alone or their combinations at different concentrations for 24 h. 2-DG was added 1 h before adding MJ or COR. Stock samples of MJ and COR were dissolved in ethanol and DMSO, respectively, and were diluted with the respective culture media immediately before performing the experiments. Final concentrations of ethanol and DMSO in the cell culture medium were always <0.1% (v/v) and had no effects on cell proliferation. Cell viability was determined at 24 h after the treatment by adding 10 μL MTT solution, followed by incubation for 1 h at 37 °C. Formazan crystals in viable cells were dissolved using 100 μL DMSO. Optical density of the dissolved formazan was measured using a microplate spectrophotometer (xMark; Bio-Rad, Berkeley, CA) at 570 nm. Cell viability data were then analyzed using CompuSyn software (ComboSyn Inc., Paramus, NJ) to calculate the IC_50_ and CI values. CI values provide a quantitative assessment of the synergism between drugs being examined based on Chou and Talalay method. CI values were calculated using the following equation: CI = (D)_1_/(D_x_)_1_ + (D)_2_/(D_x_)_2_, where (D)_1_ and (D)_2_ are the doses of drugs 1 and 2, respectively, used in combination for a given effect and (D_x_)_1_ and (D_x_)_2_ are the doses of the drugs 1 and 2 alone, respectively, inducing the given effect. CI values of 1, < 1, and >1 indicate that the drugs 1 and 2 exert additive, synergistic, and antagonistic effects, respectively^84^.

### Cell proliferation assay

The proliferation of A375 and A2058 cells (density, 2 × 10^4^ cells/well) was measured using Cell Proliferation ELISA, BrdU (colorimetric) kit (Sigma-Aldrich), according to the manufacturer’s instructions.

### Sample preparation

The cells (seeding density, 4 × 10^5^ cells/well) were detached by treating with trypsin-EDTA solution and the cells from four wells of a six-well plate were pooled. Next, the cells were pelleted by centrifugation at 1000 × *g* and 4 °C for 2 min, washed twice with ice-cold PBS to remove extracellular metabolites and residual medium, and immediately frozen with liquid nitrogen to quench cellular metabolism. The deep-frozen cells were suspended in PBS and were lysed using two freeze-thaw cycles. Briefly, the cells were thawed in a 4 °C water bath, vortexed, and sonicated on ice for 20 min. Next, the cells were transferred to liquid nitrogen for 60 min, thawed in a 37 °C water bath for 30 min, and vortexed briefly. This freeze-thaw cycle was repeated once for complete cell lysis, followed by additional sonication for 10 min. Protein content in the cell lysates was estimated using BCA Protein Assay Kit (Thermo Scientific, Rockford, IL), with bovine serum albumin as a standard for normalization. Before the analysis, the samples were freeze-dried and stored at −80 °C. Metabolites and lipids were extracted by using a modified Folch procedure, as described previously^45^. Briefly, an extraction mixture (chloroform/methanol, 2:1, v/v) was added to the freeze-dried cells. The mixture along with the cells was vortexed for 20 s, followed by sonication in ice-water for 30 min. The mixture was then incubated with shaking on ice for 40 min, followed by the addition of ice-cold water to induce phase separation. The mixture was incubated again with shaking for 10 min and was centrifuged at 18,500 × *g* and 4 °C for 10 min. Both the upper phase and lower phase were collected separately for performing metabolite and lipid analyses.

### Metabolite analysis using GC-MS

Derivatization was performed by transferring 0.4 mL cell extracts into GC vials, followed by evaporation under nitrogen gas. The dried extracts were dissolved in 30 μL 20,000 μg/mL methoxyamine hydrochloride in pyridine, 50 μL BSTFA containing 1% TMCS, and 10 μL myristic-d_27_ acid in pyridine (500 μg/mL as an internal standard) and were incubated at 65 °C for 60 min before performing the GC-MS analysis. Each derivatized sample (1 μL) was injected into Agilent 7890 A GC system equipped with a 7683B series autosampler and 5975 C mass selective detector (Agilent Technologies, Santa Clara, CA), with a split ratio of 1:10. DB5-MS column (length, 30 m; inner diameter, 0.25 mm; and film thickness, 0.25 μm; Agilent Technologies) was used with a constant flow rate of 1.0 mL/min, with helium as carrier gas. In electron impact ionization mode, electron energy was 70 eV. Temperatures of the ion source, quadrupole, and auxiliary were set to 230 °C, 150 °C, and 280 °C, respectively. A full-scan mode was used in a mass range of 50–700 Da. The GC analysis of metabolites in the cell extracts was initiated at an initial oven temperature of 70 °C, followed by an increase in the temperature to 190 °C (at 5 °C/min), 240 °C (at 6 °C/min), and 280 °C (at 5 °C/min). All the metabolites were identified by comparing the obtained mass spectra with NIST-Wiley Mass Spectra Library, and peaks with a matching quality of >70% were assigned compound names. The spectra were also matched with the spectra in the Human Metabolome Database (HMDB; http://www.hmdb.ca/) and Golm Metabolome Database (GMD; gmd.mpimp-golm.mpg.de/). Leucine and isoleucine were identified by using chemical standards to compare the retention times as well as MS/MS fragmentation.

### Lipid analysis using nanoESI-MS

Lipid extracts were passed through a 0.2-μm PTFE syringe filter (Whatman, Maidstone, UK), and 0.9 mL filtrate taken and evaporated under nitrogen gas. The dried extracts were reconstituted in 150 μL methanol/chloroform (9:1, v/v) containing 7.5 mM ammonium acetate buffer solution. 1,2-Diheptadecanoyl-sn-glycero-3-phosphoethanolamine (PE 17:0/17:0: *m/z* of 720.5 in the positive ion mode and 718.5 in the negative ion mode) was used as an internal standard. Automated shotgun experiments were performed using linear-ion-trap mass spectrometer (LTQ-XL; Thermo Fisher Scientific, San Jose, CA) equipped with a robotic nanoflow ion source (TriVersa NanoMate System; Advion Biosciences, Ithaca, NY) in the positive and negative ion modes. Next, 10 μL sample was infused into the MS system through a nanoelectrospray chip with 5.5-μm-diameter spray nozzles. The ion source was controlled using Chipsoft 8.3.1 software (Advion Biosciences). Ionization voltage and backpressure were 1.4 kV and 0.4 psi, respectively, in the positive ion mode and −1.7 kV and 0.6 psi, respectively, in the negative ion mode. Data were acquired in the profile mode for 2 min, and scan range was set at *m/z* 400–1,200. Capillary and tube lens voltages were set to 49 and 145 V, respectively, in the positive ion mode and −9 and −72.67 V, respectively, in the negative ion mode. A data-dependent MS/MS scan was performed under the collision energy offset of 35 eV. Dynamic exclusion parameters were set at a repeat duration of 60 s, exclusion duration of 60 s, and exclusion list size of 50. All spectra were recorded using Xcalibur software (version 2.2.; Thermo Fisher Scientific), and lipid species were identified using LipidMAPS (http://www.lipidmaps.org/), LipidBlast, and an in-house MS/MS library.

### Data processing

To relatively quantify the metabolites and lipids, the GC-MS spectrum data were processed using Expressionist^®^ MSX software (version 2013.0.39; GeneData, Basel, Switzerland). A list of retention time, base peak intensity, and *m/z* values was obtained for each chromatogram. Raw data files (*.raw) of lipids were converted to *.mzXML files by using ProteoWizard msConvert software, and the spectrum data were further processed using Expressionist^®^ MSX software. Data matrices, including *m/z* and peak intensity, were exported as Excel files (version 2010; Microsoft, Redmond, WA). Normalization was performed by dividing the peak intensity of each compound by that of the internal standard and by the total protein content.

### Statistical analysis

The resulting datasets were used for performing PCA and OPLS-DA by using SIMCA-P + software (version 13.0; Umetrics, Umeå, Sweden). All variables were preprocessed with mean-centered and scaled-to-pareto scaling. Significant differences were evaluated using Student’s *t*-test (at *p* < 0.05) with SPSS software (version 23; IBM, Somers, NY). Data on fold changes were assessed using a web-based data processing tool MetaboAnalyst (version 3.0; http://www.metaboanalyst.ca).

## Supplementary information

Supplementary Materials

## Data Availability

Most data generated during this study are included in this manuscript and its supplementary information files. Datasets generated during and/or analyzed in the present study can be obtained from the corresponding author upon a reasonable request.
