# Supplementary material for: Metabolic and lipidomic investigation of the antiproliferative effects of coronatine against human melanoma cells
Source: Sci Rep. 2019 Feb 28;9:3140. doi: 10.1038/s41598-019-39990-w (PMC6395766; doi:10.1038/s41598-019-39990-w)
Supplement: Supplementary file 1 — Supplementary Materials [file 41598_2019_39990_MOESM1_ESM.pdf]

Supplementary Materials

Metabolic and lipidomic investigation of the antiproliferative effects of coronatine against human melanoma cells

Hye-Youn Kim, Hanyong Jin, Jeehyeon Bae, Hyung-Kyoon Choi*

College of Pharmacy, Chung-Ang University, Seoul 06974, Republic of Korea

*To whom correspondence should be addressed

Tel: +82-2-820-5605

Fax: +82-2-812-3912

E-mail: hykychoi@cau.ac.kr

Supplementary Table S1. Identification of metabolites from melanoma cells and human epidermal melanocytes using GC-MS. RT, retention time; TMS, trimethylsilylation.

Compound	RT (min)	Fragmentation ion (m/z)	TMS
**Alcohol**			
Myo-inositol	28.88	191, 217, **305**, 318	6
Glucitol	26.21	103, 205, 517, **319**	6
**Amino acid**			
Alanine	6.99	100, **116**, 190, 218	2
β-Alanine	15.07	133, **174**, 248, 290	3
Aspartic acid	17.37	100, 202, 218, **232**	3
Cysteine	18.16	100, 132, **220**, 294	3
Glutamic acid	19.72	128, 156, **246**, 348	3
Glycine	7.45	59, **102**, 176, 204	2
	11.97	86, **174**, 248, 276	3
Isoleucine	8.80	75, **86**, 146, 188	1
	11.67	**158**, 218, 232, 260	2
Leucine	8.28	75, **86**, 146, 188	1
Lysine	26.01	128, 156, **174**, 317	4
Proline	8.74	**70**, 75, 103, 172	1
Pyroglutamic acid	17.32	84, **156**, 230, 258	2
Serine	10.77	103, **116**, 132, 234	2
	13.40	100, 133, **204**, 218	3
Threonine	11.70	**117**, 130, 219, 248	2
	14.03	101, 117, **218**, 291	3
Tyrosine	26.32	100, 179, **218**, 280	3
Valine	6.71	55, **72**, 130, 146	1
	9.70	100, 133, **144**, 218	2
**Organic acid**			
Aminomalonic acid	16.10	133, 174, **218**, 320	3
Fumaric acid	13.22	133, 155, 217, **245**	2
Lactic acid	6.02	**117**, 133, 191, 219	2
Malic acid	16.61	133, **233**, 245, 335	3
**Purine**			
Hypoxanthine	23.71	193, 206, **265**, 280	2
Inosine	36.07	**217**, 230, 245, 281	4

Guanine	29.55	99, 280, **352**, 367	3
**Pyrimidine**			
Uracil	12.81	99, 113, **241**, 256	2
Uridine	34.28	103, 169, **217**, 259	3
**Sugar**			
Glucose	27.09	129, 191, **204**, 217	5
Glyceric acid	12.63	133, **189**, 292, 307	3
Glucose-6-phosphate	33.45	204, 299, 357, **387**	6
Mannose-6-phosphate	32.26	217, 299, 357, **387**	6(1MEOX)
Ribose	20.97	**103**, 189, 217, 307	4(1MEOX)
**Other**			
Creatinine	18.04	100, **115**, 143, 329	3
Phosphoric acid	22.82	103, 299, **357**, 445	4

Supplementary Table S2. Fold-changes and *p*-values of metabolites identified from melanoma cells (A375 and A2058) and human epidermal melanocytes (HEMn-LP) by GC-MS. The log2(FC) presents the $\log_2$ ratio of peak intensity of metabolites in COR-treated group versus untreated groups. Positive values of log2(FC) indicate an increase in the COR-treated groups, whereas negative values indicate a decrease. The *p*-values were obtained by the Student's *t*-test. ND, not detected; ns, not significant difference ($p > 0.05$); ↑, significantly increased level compared with control ($p < 0.05$); ↓, significantly decreased level compared with control ($p < 0.05$).

Compound	HEMn-LP				A375				A2058			
	1.0 µM COR		2-DG + 0.01 µM COR		1.0 µM COR		2-DG + 0.01 µM COR		1.0 µM COR		2-DG + 0.01 µM COR	
	p	log2(FC)	*p*	log2(FC)	*p*	log2(FC)	*p*	log2(FC)	*p*	log2(FC)	*p*	log2(FC)
**Alcohol**												
Myo-inositol	0.030	0.40 (↑)	0.707	-0.03 (ns)	0.042	-0.11 (↓)	0.146	-0.07 (ns)	0.000	-0.43 (↓)	0.000	-0.23 (↓)
Glucitol	ND	ND	ND	ND	0.578	-0.03 (ns)	0.005	-0.30 (↓)	0.001	-0.77 (↓)	0.019	-0.38 (↓)
**Amino acid**												
Alanine	0.026	0.21 (↑)	0.054	-0.15 (ns)	0.003	-0.28 (↓)	0.004	-0.16 (↓)	0.003	-0.45 (↓)	0.002	-0.49 (↓)
β-Alanine	0.537	0.06 (ns)	0.018	-0.45 (↓)	0.012	-0.13 (↓)	0.004	-0.19 (↓)	0.023	0.27 (↑)	0.004	0.42 (↑)
Aspartic acid	0.586	0.05 (ns)	0.000	-0.91 (↓)	0.000	-0.78 (↓)	0.000	-1.10 (↓)	0.038	-0.74 (↓)	0.019	-0.88 (↓)
Cysteine	0.009	0.44 (↑)	0.900	0.02 (ns)	0.008	-0.28 (↓)	0.000	0.54 (↑)	0.000	-1.31 (↓)	0.000	-1.08 (↓)
Glutamic acid	0.023	0.22 (↑)	0.049	-0.17 (↓)	0.001	-0.16 (↓)	0.000	-0.18 (↓)	0.001	-0.26 (↓)	0.000	-0.43 (↓)
Glycine	0.988	-5.4E-04 (ns)	0.000	-0.47 (↓)	0.000	-0.13 (↓)	0.179	0.04 (ns)	0.146	-0.15 (ns)	0.106	-0.14 (ns)
Isoleucine	0.187	-0.05 (ns)	0.000	-0.32 (↓)	0.014	-0.16 (↓)	0.000	0.58 (↑)	0.002	-0.35 (↓)	0.007	-0.49 (↓)
Leucine	0.369	0.09 (ns)	0.209	-0.12 (ns)	0.629	-0.05 (ns)	0.001	0.74 (↑)	0.653	-0.04 (ns)	0.042	-0.23 (↓)
Lysine	0.140	-0.30 (ns)	0.007	-0.82 (↓)	0.000	-0.45 (↓)	0.000	0.78 (↑)	0.048	0.43 (↑)	0.362	-0.2 (ns)
Proline	0.008	0.27 (↑)	0.679	0.04 (ns)	0.008	-0.35 (↓)	0.034	-0.35 (↓)	0.002	-0.59 (↓)	0.001	-0.78 (↓)

Pyroglutamic acid	0.080	0.16 (ns)	0.270	-0.07 (ns)	0.006	-0.19 (↓)	0.000	0.23 (↑)	0.058	0.14 (ns)	0.111	0.13 (ns)
Serine	0.001	-0.34 (↓)	0.000	-0.89 (↓)	0.002	-0.32 (↓)	0.009	0.18 (↑)	0.001	-0.43 (↓)	0.001	-0.62 (↓)
Threonine	0.024	-0.24 (↓)	0.000	-0.78 (↓)	0.000	-0.30 (↓)	0.008	-0.22 (↓)	0.001	-0.45 (↓)	0.000	-0.53 (↓)
Tyrosine	ND	ND	ND	ND	0.000	-0.45 (↓)	0.000	0.38 (↑)	0.019	0.40 (↑)	0.333	-0.16 (ns)
Valine	0.081	-0.10 (ns)	0.001	-0.38 (↓)	0.002	-0.20 (↓)	0.001	0.30 (↑)	0.000	-0.36 (↓)	0.000	-0.48 (↓)
**Organic acid**												
Aminomalonic acid	0.599	-0.04 (ns)	0.002	-0.57 (↓)	0.018	-0.22 (↓)	0.001	-0.30 (↓)	0.012	-0.43 (↓)	0.021	-0.38 (↓)
Fumaric acid	0.002	0.30 (↑)	0.069	-0.14 (ns)	0.014	-0.15 (↓)	0.000	-0.48 (↓)	0.005	-0.76 (↓)	0.005	-0.71 (↓)
Lactic acid	0.161	-0.07 (ns)	0.034	-0.09 (↓)	0.004	-0.27 (↓)	0.000	-0.46 (↓)	0.001	-0.52 (↓)	0.002	-0.48 (↓)
Malic acid	0.013	0.19 (↑)	0.001	-0.38 (↓)	0.006	-0.20 (↓)	0.001	-0.52 (↓)	0.000	-0.57 (↓)	0.000	-0.44 (↓)
**Purine**												
Hypoxanthine	0.408	-0.12 (ns)	0.003	-1.20 (↓)	0.000	-0.37 (↓)	0.000	-0.68 (↓)	0.890	0.03 (ns)	0.922	0.02 (ns)
Inosine	0.015	-0.46 (↓)	0.002	-0.69 (↓)	0.014	-0.18 (↓)	0.000	-0.94 (↓)	0.110	-0.54 (ns)	0.777	0.06 (ns)
Guanine	ND	ND	ND	ND	0.477	0.05 (ns)	0.713	-0.03 (ns)	0.832	0.08 (ns)	0.977	0.01 (ns)
**Pyrimidine**												
Uracil	0.007	0.30 (↑)	0.350	0.07 (ns)	0.000	-0.15 (↓)	0.000	-1.25 (↓)	0.000	-0.95 (↓)	0.455	-0.08 (ns)
Uridine	0.124	-0.09 (ns)	0.045	-0.31 (↓)	0.043	-0.14 (↓)	0.009	-0.27 (↓)	0.003	-0.68 (↓)	0.001	-0.95 (↓)
**Sugar**												
Glucose	0.100	-0.17 (ns)	0.014	-0.36 (↓)	0.000	-0.27 (↓)	0.000	-1.18 (↓)	0.000	-0.50 (↓)	0.001	-0.32 (↓)
Glyceric acid	ND	ND	ND	ND	0.002	-0.40 (↓)	0.000	-1.15 (↓)	0.000	-1.51 (↓)	0.000	-0.82 (↓)
Glucose-6-phosphate	0.439	0.07 (ns)	0.064	-0.14 (ns)	0.004	-0.48 (↓)	0.002	-0.67 (↓)	0.006	-0.57 (↓)	0.015	-0.42 (↓)
Mannose-6-phosphate	0.000	-0.59 (↓)	0.015	-0.30 (↓)	0.003	-0.40 (↓)	0.001	-0.67 (↓)	0.002	-0.61 (↓)	0.003	-0.60 (↓)

Ribose	ND	ND	ND	ND	0.003	-0.24 (↓)	0.000	-0.62 (↓)	0.000	-0.86 (↓)	0.000	-0.45 (↓)
**Other**												
Creatinine	0.483	-0.15 (ns)	0.024	-0.64 (↓)	0.056	-0.13 (ns)	0.001	-0.40 (↓)	0.000	-0.60 (↓)	0.000	-0.69 (↓)
Phosphoric acid	0.107	0.12 (ns)	0.002	-0.37 (↓)	0.038	-0.07 (↓)	0.001	1.85 (↑)	0.674	-0.03 (ns)	0.000	-1.29 (↓)

Supplementary Table S3. MS/MS fragment ion of identified lipid species from extracts of human melanocytes and melanoma cell lines.

Lipid molecular species	Proposed composition	Ion species	m/z	MS/MS fragment ion (m/z)
**Positive ion mode**				
Phosphatidylcholine (PC)				
PC 30:0	C14:0/C16:0	$[M + H]^+$	706	**450** [lyso-PC(14:0) – H_2O + H]$^+$; **468** [lyso-PC(14:0) + H]$^+$; **478** [lyso-PC(16:0) – H_2O + H]$^+$; **496** [lyso-PC(16:0) + H]$^+$; **523** [M – $C_5H_{14}NO_4P$ + H]$^+$; **647** [M – C_3H_9N + H]$^+$; **688** [M – H_2O + H]$^+$
PC 32:1	C16:0/C16:1	$[M + H]^+$	732	**476** [lyso-PC(16:1) – H_2O + H]$^+$; **478** [lyso-PC(16:0) – H_2O + H]$^+$; **494** [lyso-PC(16:1) + H]$^+$; **496** [lyso-PC(16:0) + H]$^+$; **549** [M – $C_5H_{14}NO_4P$ + H]$^+$; **673** [M – C_3H_9N + H]$^+$; **714** [M – H_2O + H]$^+$
PC 32:0	C16:0/C16:0	$[M + H]^+$	734	**478** [lyso-PC(16:0) – H_2O + H]$^+$; **496** [lyso-PC(16:0) + H]$^+$; **551** [M – $C_5H_{14}NO_4P$ + H]$^+$; **675** [M – C_3H_9N + H]$^+$; **716** [M – H_2O + H]$^+$
plasmenyl-PC 32:0	C16:0/C16:0	$[M + Na]^+$	740	**279** [(M + Na) – $C_5H_{14}NO_4P$ – Na – C16:0]$^+$; **535** [(M + Na) – $C_5H_{14}NO_4P$ – Na + H]$^+$; **557** [(M + Na) – $C_5H_{14}NO_4P$]$^+$; **681** [(M + Na) – C_3H_9N]$^+$
PC 34:2	C16:1/C18:1	$[M + H]^+$	758	**476** [lyso-PC(16:1) – H_2O + H]$^+$; **494** [lyso-PC(16:1) + H]$^+$; **504** [lyso-PC(18:1) – H_2O + H]$^+$; **522** [lyso-PC(18:1) + H]$^+$; **575** [M – $C_5H_{14}NO_4P$ + H]$^+$; **699** [M – C_3H_9N + H]$^+$; **740** [M – H_2O + H]$^+$
PC 34:1	C16:0/C18:1	$[M + H]^+$	760	**478** [lyso-PC(16:0) – H_2O + H]$^+$; **496** [lyso-PC(16:0) + H]$^+$; **504** [lyso-PC(18:1) – H_2O + H]$^+$; **522** [lyso-PC(18:1) + H]$^+$; **577** [M – $C_5H_{14}NO_4P$ + H]$^+$; **701** [M – C_3H_9N + H]$^+$; **742** [M – H_2O + H]$^+$
PC 34:0	C16:0/C18:0	$[M + H]^+$	762	**478** [lyso-PC(16:0) – H_2O + H]$^+$; **496** [lyso-PC(16:0) + H]$^+$; **506** [lyso-PC(18:0) – H_2O + H]$^+$; **524** [lyso-PC(18:0) + H]$^+$; **579** [M – $C_5H_{14}NO_4P$ + H]$^+$; **703** [M – C_3H_9N + H]$^+$; **744** [M – H_2O + H]$^+$
plasmenyl-PC 34:0	C16:0/C18:0	$[M + Na]^+$	768	**279** [(M + Na) – $C_5H_{14}NO_4P$ – Na – C18:0]$^+$; **563** [(M + Na) – $C_5H_{14}NO_4P$ – Na + H]$^+$; **585** [(M + Na) – $C_5H_{14}NO_4P$]$^+$; **709** [(M + Na) – C_3H_9N]$^+$
PC 36:2	C18:1/C18:1	$[M + H]^+$	786	**504** [lyso-PC(18:1) – H_2O + H]$^+$; **522** [lyso-PC(18:1) + H]$^+$; **603** [M – $C_5H_{14}NO_4P$ + H]$^+$; **727** [M – C_3H_9N + H]$^+$; **768** [M – H_2O + H]$^+$
PC 36:1	C18:0/C18:1	$[M + H]^+$	788	**504** [lyso-PC(18:1) – H_2O + H]$^+$; **506** [lyso-PC(18:0) – H_2O + H]$^+$; **522** [lyso-PC(18:1) + H]$^+$; **524** [lyso-PC(18:0) + H]$^+$; **605** [M – $C_5H_{14}NO_4P$ + H]$^+$; **729** [M – C_3H_9N + H]$^+$; **770** [M – H_2O + H]$^+$
PC 36:3	C18:1/C18:2	$[M + Na]^+$	806	**465** [(M + Na) – C_3H_9N – C18:1]$^+$; **467** [(M + Na) – C_3H_9N – C18:2]$^+$; **524** [(M + Na) – C18:1 – H_2O]$^+$; **526** [(M + Na) – C18:2 – H_2O]$^+$; **623** [(M + Na) – $C_5H_{14}NO_4P$]$^+$; **747** [(M +

				Na) – C_3H_9N]$^+$
Triacylglycerol (TG)				
TG 50:2	C16:0/C16:1/C18:1	[M + NH_4]$^+$	848	**549** [M + NH_4 – C18:1 – 18]$^+$; **575** [M + NH_4 – C16:0– 18]$^+$; **577** [M + NH_4 – C16:1– 18]$^+$; **831** [M + NH_4 – 17]$^+$
TG 50:1	C16:0/C16:1/C18:0	[M + NH_4]$^+$	850	**549** [M + NH_4 – C18:0 – 18]$^+$; **577** [M + NH_4 – C16:0– 18]$^+$; **579** [M + NH_4 – C16:1– 18]$^+$; **833** [M + NH_4 – 17]$^+$
TG 52:3	C16:1/C18:1/C18:1	[M + NH_4]$^+$	874	**575** [M + NH_4 – C18:1 – 18]$^+$; **603** [M + NH_4 – C16:1– 18]$^+$; **857** [M + NH_4 – 17]$^+$
TG 52:2	C16:0/C18:1/C18:1	[M + NH_4]$^+$	876	**577** [M + NH_4 – C18:1 – 18]$^+$; **603** [M + NH_4 – C16:0– 18]$^+$; **859** [M + NH_4 – 17]$^+$
TG 52:1	C16:0/C18:0/C18:1	[M + NH_4]$^+$	878	**577** [M + NH_4 – C18:0 – 18]$^+$; **579** [M + NH_4 – C18:1 – 18]$^+$; **605** [M + NH_4 – C16:0– 18]$^+$; **861** [M + NH_4 – 17]$^+$
TG 54:3	C18:1/C18:1/C18:1	[M + NH_4]$^+$	902	**603** [M + NH_4 – C18:1 – 18]$^+$; **885** [M + NH_4 – 17]$^+$
**Negative ion mode**				
Ceramide (Cer)				
Cer d34:2	C18:1/C16:1	[M − H]$^-$	534	**235** [M – H – CH_2O – $C_{17}H_{35}ON$]$^-$; **252** [M – H – H_2O – $C_{18}H_{32}O$]$^-$; **253** [M – H – H_2O – $C_{18}H_{33}N$]$^-$; **278** [M – H – $C_{16}H_{30}O$ – H_2O]$^-$; **294** [M – H – $C_{16}H_{32}O$]$^-$; **502** [M – H – CH_3OH]$^-$; **504** [M – H – CH_2O]$^-$; **516** [M – H – H_2O]$^-$
Cer d34:1	C18:1/C16:0	[M − H]$^-$	536	**237** [M – H – CH_2O – $C_{17}H_{35}ON$]$^-$; **254** [M – H – H_2O – $C_{18}H_{32}O$]$^-$; **255** [M – H – H_2O – $C_{18}H_{33}N$]$^-$; **280** [M – H – $C_{16}H_{30}O$ – H_2O]$^-$; **296** [M – H – $C_{16}H_{32}O$]$^-$; **488** [M – H – CH_2O – H_2O]$^-$; **504** [M – H – CH_3OH]$^-$; **506** [M – H – CH_2O]$^-$; **518** [M – H – H_2O]$^-$
Cer d36:2	C18:1/C18:1	[M − H]$^-$	562	**262** [M – H – CH_2O – $C_{17}H_{35}ON$]$^-$; **281** [M – H – H_2O – $C_{18}H_{33}N$]$^-$; **306** [M – H – $C_{16}H_{30}O$ – H_2O]$^-$; **322** [M – H – $C_{16}H_{32}O$]$^-$; **514** [M – H – CH_2O – H_2O]$^-$; **530** [M – H – CH_3OH]$^-$; **532** [M – H – CH_2O]$^-$; **544** [M – H – H_2O]$^-$
Cer d36:1	C18:1/C18:0	[M − H]$^-$	564	**265** [M – H – CH_2O – $C_{17}H_{35}ON$]$^-$; **282** [M – H – H_2O – $C_{18}H_{32}O$]$^-$; **283** [M – H – H_2O – $C_{18}H_{33}N$]$^-$; **308** [M – H – $C_{16}H_{30}O$ – H_2O]$^-$; **324** [M – H – $C_{16}H_{32}O$]$^-$; **516** [M – H – CH_2O – H_2O]$^-$; **532** [M – H – CH_3OH]$^-$; **534** [M – H – CH_2O]$^-$; **546** [M – H – H_2O]$^-$
Cer d36:0	C18:0/C18:0	[M − H]$^-$	566	**265** [M – H – CH_2O – $C_{17}H_{37}ON$]$^-$; **282** [M – H – H_2O – $C_{18}H_{34}O$]$^-$; **283** [M – H – H_2O – $C_{18}H_{35}N$]$^-$; **309** [M – H – $C_{16}H_{32}O$ – H_2O]$^-$; **324** [M – H – $C_{16}H_{34}O$]$^-$; **518** [M – H – CH_2O – H_2O]$^-$; **534** [M – H – CH_3OH]$^-$; **536** [M – H – CH_2O]$^-$; **548** [M – H – H_2O]$^-$
Cer d38:2	C18:1/C20:1	[M − H]$^-$	590	**291** [M – H – CH_2O – $C_{17}H_{35}ON$]$^-$; **308** [M – H – H_2O – $C_{18}H_{32}O$]$^-$; **309** [M – H – H_2O – $C_{18}H_{33}N$]$^-$; **334** [M – H – $C_{16}H_{30}O$ – H_2O]$^-$; **350** [M – H – $C_{16}H_{32}O$]$^-$; **542** [M – H – CH_2O –

				H_2O]$^-$; **558** [M – H – CH_3OH]$^-$; **560** [M – H – CH_2O]$^-$; **572** [M – H – H_2O]$^-$
Cer d38:1	C18:1/C20:0	$[M - H]^-$	592	**293** [M – H – CH_2O – $C_{17}H_{35}ON$]$^-$; **310** [M – H – H_2O – $C_{18}H_{32}O$]$^-$; **311** [M – H – H_2O – $C_{18}H_{33}N$]$^-$; **336** [M – H – $C_{16}H_{30}O$ – H_2O]$^-$; **352** [M – H – $C_{16}H_{32}O$]$^-$; **544** [M – H – CH_2O – H_2O]$^-$; **560** [M – H – CH_3OH]$^-$; **562** [M – H – CH_2O]$^-$; **574** [M – H – H_2O]$^-$
Cer d38:0	C18:0/C20:0	$[M - H]^-$	594	**311** [M – H – H_2O – $C_{18}H_{35}N$]$^-$; **336** [M – H – $C_{16}H_{32}O$ – H_2O]$^-$; **352** [M – H – $C_{16}H_{34}O$]$^-$; **546** [M – H – CH_2O – H_2O]$^-$; **562** [M – H – CH_3OH]$^-$; **564** [M – H – CH_2O]$^-$; **576** [M – H – H_2O]$^-$
Cer d40:1	C18:1/C22:0	$[M - H]^-$	620	**321** [M – H – CH_2O – $C_{17}H_{35}ON$]$^-$; **338** [M – H – H_2O – $C_{18}H_{32}O$]$^-$; **339** [M – H – H_2O – $C_{18}H_{33}N$]$^-$; **364** [M – H – $C_{16}H_{30}O$ – H_2O]$^-$; **380** [M – H – $C_{16}H_{32}O$]$^-$; **572** [M – H – CH_2O – H_2O]$^-$; **588** [M – H – CH_3OH]$^-$; **590** [M – H – CH_2O]$^-$; **602** [M – H – H_2O]$^-$
Phosphatidylethanolamine (PE)				
plasmenyl-PE 34:1	C16:0/C18:1	$[M - H]^-$	700	**281** [C18:1 – H]$^-$; **418** [lyso-PE(P-16:0) – H_2O – H]$^-$; **436** [lyso-PE(P-16:0) – H]$^-$
PE 34:2	C16:1/C18:1	$[M - H]^-$	714	**253** [C16:1 – H]$^-$; **281** [C18:1 – H]$^-$; **432** [lyso-PE(16:1) – H_2O – H]$^-$; **450** [lyso-PE(16:1) – H]$^-$; **460** [lyso-PE(18:1) – H_2O – H]$^-$; **478** [lyso-PE(18:1) – H]$^-$
PE 34:1	C16:0/C18:1	$[M - H]^-$	716	**255** [C16:0 – H]$^-$; **281** [C18:1 – H]$^-$; **434** [lyso-PE(16:0) – H_2O – H]$^-$; **452** [lyso-PE(16:0) – H]$^-$; **460** [lyso-PE(18:1) – H_2O – H]$^-$; **478** [lyso-PE(18:1) – H]$^-$
plasmenyl-PE 36:4	C16:0/C20:4	$[M - H]^-$	722	**303** [C20:4 – H]$^-$; **418** [lyso-PE(P-16:0) – H_2O – H]$^-$; **436** [lyso-PE(P-16:0) – H]$^-$
PE 36:2	C18:1/C18:1	$[M - H]^-$	742	**281** [C18:1 – H]$^-$; **460** [lyso-PE(18:1) – H_2O – H]$^-$; **478** [lyso-PE(18:1) – H]$^-$
PE 36:1	C18:0/C18:1	$[M - H]^-$	744	**281** [C18:1 – H]$^-$; **283** [C18:0 – H]$^-$; **460** [lyso-PE(18:1) – H_2O – H]$^-$; **462** [lyso-PE(18:0) – H_2O – H]$^-$; **478** [lyso-PE(18:1) – H]$^-$; **480** [lyso-PE(18:0) – H]$^-$
plasmenyl-PE 38:6	C16:0/C22:6	$[M - H]^-$	746	**327** [C22:6 – H]$^-$; **418** [lyso-PE(P-16:0) – H_2O – H]$^-$; **436** [lyso-PE(P-16:0) – H]$^-$
plasmenyl-PE 38:5	C16:0/C22:5	$[M - H]^-$	748	**329** [C22:5 – H]$^-$; **418** [lyso-PE(P-16:0) – H_2O – H]$^-$; **436** [lyso-PE(P-16:0) – H]$^-$
plasmenyl-PE 38:4	C18:0/C20:4	$[M - H]^-$	750	**303** [C20:4 – H]$^-$; **446** [lyso-PE(P-18:0) – H_2O – H]$^-$; **464** [lyso-PE(P-18:0) – H]$^-$
PE 38:5	C18:1/C20:4	$[M - H]^-$	764	**281** [C18:1 – H]$^-$; **303** [C20:4 – H]$^-$; **460** [lyso-PE(18:1) – H_2O – H]$^-$; **478** [lyso-PE(18:1) – H]$^-$; **482** [lyso-PE(20:4) – H_2O – H]$^-$; **500** [lyso-PE(20:4) – H]$^-$
PE 38:4	C18:0/C20:4	$[M - H]^-$	766	**283** [C18:0 – H]$^-$; **303** [C20:4 – H]$^-$; **462** [lyso-PE(18:0) – H_2O – H]$^-$; **480** [lyso-PE(18:0) – H]$^-$; **482** [lyso-PE(20:4) – H_2O – H]$^-$; **500** [lyso-PE(20:4) – H]$^-$
plasmenyl-PE 40:5	C18:0/C22:5	$[M - H]^-$	776	**329** [C22:5 – H]$^-$; **446** [lyso-PE(P-18:0) – H_2O – H]$^-$; **464** [lyso-PE(P-18:0) – H]$^-$
plasmenyl-PE 40:4	C18:0/C22:4	$[M - H]^-$	778	**331** [C22:4 – H]$^-$; **446** [lyso-PE(P-18:0) – H_2O – H]$^-$; **464** [lyso-PE(P-18:0) – H]$^-$

PE 40:5	C18:0/C22:5	$[M-H]^-$	792	**283** [C18:0 – H]⁻; **329** [C22:5 – H]⁻; **462** [lyso-PE(18:0) – H_2O – H]⁻; **480** [lyso-PE(18:0) – H]⁻; **508** [lyso-PE(22:5) – H_2O – H]⁻; **526** [lyso-PE(22:5) – H]⁻
PE 40:4	C18:0/C22:4	$[M-H]^-$	794	283 [C18:0 – H]⁻; 331 [C22:4 – H]⁻; 462 [lyso-PE(18:0) – H_2O – H]⁻; 480 [lyso-PE(18:0) – H]⁻; 510 [lyso-PE(22:4) – H_2O – H]⁻; 528 [lyso-PE(22:4) – H]⁻
Phosphatidylglycerol (PG)				
PG 34:1	C16:0/C18:1	$[M-H]^-$	747	**255** [C16:0 – H]⁻; **281** [C18:1 – H]⁻; **391** [lyso-PG(16:0) – $C_3H_6O_2$ – H]⁻; **417** [lyso-PG(18:1) – $C_3H_6O_2$ – H]⁻; **465** [lyso-PG(16:0) – H_2O – H]⁻; **483** [lyso-PG(16:0) – H]⁻; **491** [lyso-PG(18:1) – H_2O – H]⁻; **509** [lyso-PG(18:1) – H]⁻
PG 36:2	C18:1/C18:1	$[M-H]^-$	773	**281** [C18:1 – H]⁻; **417** [lyso-PG(18:1) – $C_3H_6O_2$ – H]⁻; **491** [lyso-PG(18:1) – H_2O – H]⁻; **509** [lyso-PG(18:1) – H]⁻
PG 36:1	C18:0/C18:1	$[M-H]^-$	775	**281** [C18:1 – H]⁻; **283** [C18:0 – H]⁻; **417** [lyso-PG(18:1) – $C_3H_6O_2$ – H]⁻; **419** [lyso-PG(18:0) – $C_3H_6O_2$ – H]⁻; **491** [lyso-PG(18:1) – H_2O – H]⁻; **493** [lyso-PG(18:0) – H_2O – H]⁻; **509** [lyso-PG(18:1) – H]⁻; **511** [lyso-PG(18:0) – H]⁻
Phosphatidylserine (PS)				
PS 30:0	C14:0/C16:0	$[M-H]^-$	706	**227** [C14:0 – H]⁻; **255** [C16:0 – H]⁻; **363** [lyso-PS(14:0) – $C_3H_5NO_2$ – H]⁻ ; **381** [lyso-PS(14:0) – $C_3H_5NO_2$ + H_2O – H]⁻; **391** [lyso-PS(16:0) – $C_3H_5NO_2$ – H]⁻ ; **409** [lyso-PS(16:0) – $C_3H_5NO_2$ + H_2O – H]⁻; **619** [M – $C_3H_5NO_2$ – H]⁻
PS 34:2	C16:1/C18:1	$[M-H]^-$	758	**253** [C16:1 – H]⁻; **281** [C18:1 – H]⁻; **389** [lyso-PS(16:1) – $C_3H_5NO_2$ – H]⁻; **407** [lysoPS(16:1) – $C_3H_5NO_2$ + H_2O – H]⁻; **417** [lyso-PS(18:1) – $C_3H_5NO_2$ – H]⁻; **435** [lyso-PS(18:1) – $C_3H_5NO_2$ + H_2O – H]⁻; **671** [M – $C_3H_5NO_2$ – H]⁻
PS 34:1	C 16:0/C18:1	$[M-H]^-$	760	**225** [C16:0 – H]⁻; **281** [C18:1 – H]⁻; **391** [lyso-PS(16:0) – $C_3H_5NO_2$ – H]⁻ ; **409** [lyso-PS(16:0) – $C_3H_5NO_2$ + H_2O – H]⁻; **417** [lyso-PS(18:1) – $C_3H_5NO_2$ – H]⁻; **435** [lyso-PS(18:1) – $C_3H_5NO_2$ + H_2O – H]⁻; **673** [M – $C_3H_5NO_2$ – H]⁻
PS 34:0	C16:0/C18:0	$[M-H]^-$	762	**255** [C16:0 – H]⁻; **283** [C18:0 – H]⁻; **391** [lyso-PS(16:0) – $C_3H_5NO_2$ – H]⁻ ; **409** [lyso-PS(16:0) – $C_3H_5NO_2$ + H_2O – H]⁻; **419** [lyso-PS(18:0) – $C_3H_5NO_2$ – H]⁻; **437** [lyso-PS(18:0) – $C_3H_5NO_2$ + H_2O – H]⁻; **675** [M – $C_3H_5NO_2$ – H]⁻
PS 36:3	C18:1/C18:2	$[M-H]^-$	784	**279** [C18:2 – H]⁻; **281** [C18:1 – H]⁻; **415** [lyso-PS(18:2) – $C_3H_5NO_2$ – H]⁻ ; **417** [lyso-PS(18:1) $C_3H_5NO_2$ – H]⁻; **433** [lyso-PS(18:2) – $C_3H_5NO_2$ + H_2O – H]⁻; **435** [lyso-PS(18:1) – $C_3H_5NO_2$ + H_2O – H]⁻; **697** [M – $C_3H_5NO_2$ – H]⁻
PS 36:2	C18:1/C18:1	$[M-H]^-$	786	**281** [C18:1 – H]⁻; **417** [lyso-PS(18:1) – $C_3H_5NO_2$ – H]⁻; **435** [lyso-PS(18:1) – $C_3H_5NO_2$ + H_2O – H]⁻; **699** [M – $C_3H_5NO_2$ – H]⁻

PS 36:1	C18:0/C18:1	$[M - H]^-$	788	**281** [C18:1 – H]$^-$; **283** [C18:0 – H]$^-$; **417** [lyso-PS(18:1) $C_3H_5NO_2$ – H]$^-$; **419** [lyso-PS(18:0) – $C_3H_5NO_2$ – H]$^-$; **435** [lyso-PS(18:1) – $C_3H_5NO_2$ + H_2O – H]$^-$; **437** [lyso-PS(18:0)) – $C_3H_5NO_2$ + H_2O – H]$^-$; **701** [M – $C_3H_5NO_2$ – H]$^-$
PS 36:0	C18:0/C18:0	$[M - H]^-$	790	**283** [C18:0 – H]$^-$; **419** [lyso-PS(18:0) – $C_3H_5NO_2$ – H]$^-$; **437** [lyso-PS(18:0) – $C_3H_5NO_2$ + H_2O – H]$^-$; **703** [M – $C_3H_5NO_2$ – H]$^-$
PS 37:1	C18:1/C19:0	$[M - H]^-$	802	**281** [C18:1 – H]$^-$; **297** [C19:0 – H]$^-$; 417 [lyso-PS(18:1) – $C_3H_5NO_2$ – H]$^-$; **433** [lyso-PS(19:0) – $C_3H_5NO_2$ – H]$^-$; **435** [lyso-PS(18:1) – $C_3H_5NO_2$ + H_2O – H]$^-$; **451** [lyso-PS(19:0) – $C_3H_5NO_2$ + H_2O – H]$^-$; **715** [M – $C_3H_5NO_2$ – H]$^-$
PS 38:6	C16:0/C22:6	$[M - H]^-$	806	**255** [C16:0 – H]$^-$; **327** [C22:6 – H]$^-$; **391** [lyso-PS(16:0) – $C_3H_5NO_2$ – H]$^-$; **409** [lyso-PS(16:0) – $C_3H_5NO_2$ + H_2O – H]$^-$; **463** [lyso-PS(22:6) – $C_3H_5NO_2$ – H]$^-$; **481** [lyso-PS(22:6) – $C_3H_5NO_2$ + H_2O – H]$^-$; **719** [M – $C_3H_5NO_2$ – H]$^-$
PS 38:5	C18:1/C20:4	$[M - H]^-$	808	**281** [C18:1 – H]$^-$; **303** [C20:4 – H]$^-$; **417** [lyso-PS(18:1) – $C_3H_5NO_2$ – H]$^-$; **435** [lyso-PS(18:1) – $C_3H_5NO_2$ + H_2O – H]$^-$; **439** [lyso-PS(20:4) – $C_3H_5NO_2$ – H]$^-$; **457** [lyso-PS(20:4) – $C_3H_5NO_2$ + H_2O – H]$^-$; **721** [M – $C_3H_5NO_2$ – H]$^-$
PS 38:4	C18:0/C20:4	$[M - H]^-$	810	**283** [C18:0 – H]$^-$; **303** [C20:4 – H]$^-$; **419** [lyso-PS(18:0) – $C_3H_5NO_2$ – H]$^-$; **437** [lyso-PS(18:0) – $C_3H_5NO_2$ + H_2O – H]$^-$; **439** [lyso-PS(20:4) – $C_3H_5NO_2$ – H]$^-$; **457** [lyso-PS(20:4) – $C_3H_5NO_2$ + H_2O – H]$^-$; **723** [M – $C_3H_5NO_2$ – H]$^-$
PS 38:3	C18:0/C20:3	$[M - H]^-$	812	**283** [C18:0 – H]$^-$; **305** [C20:3 – H]$^-$; **419** [lyso-PS(18:0) – $C_3H_5NO_2$ – H]$^-$; **437** [lyso-PS(18:0) – $C_3H_5NO_2$ + H_2O – H]$^-$; **441** [lyso-PS(20:3) – $C_3H_5NO_2$ – H]$^-$; **459** [lyso-PS(20:3) – $C_3H_5NO_2$ + H_2O – H]$^-$; **725** [M – $C_3H_5NO_2$ – H]$^-$
PS 38:2	C18:1/C20:1	$[M - H]^-$	814	**281** [C18:1 – H]$^-$; **309** [C20:1 – H]$^-$; **417** [lyso-PS(18:1) – $C_3H_5NO_2$ – H]$^-$; **435** [lyso-PS(18:1) – $C_3H_5NO_2$ + H_2O – H]$^-$; **445** [lyso-PS(20:1) – $C_3H_5NO_2$ – H]$^-$; **463** [lyso-PS(20:1) – $C_3H_5NO_2$ + H_2O – H]$^-$; **727** [M – $C_3H_5NO_2$ – H]$^-$
PS 38:1	C18:1/C20:0	$[M - H]^-$	816	**281** [C18:1 – H]$^-$; **311** [C20:0 – H]$^-$; **417** [lyso-PS(18:1) – $C_3H_5NO_2$ – H]$^-$; **435** [lyso-PS(18:1) – $C_3H_5NO_2$ + H_2O – H]$^-$; **447** [lyso-PS(20:0) – $C_3H_5NO_2$ – H]$^-$; **465** [lyso-PS(20:0) – $C_3H_5NO_2$ + H_2O – H]$^-$; **729** [M – $C_3H_5NO_2$ – H]$^-$
PS 40:7	C18:1/C22:6	$[M - H]^-$	832	**281** [C18:1 – H]$^-$; **327** [C22:6 – H]$^-$; **417** [lyso-PS(18:1) – $C_3H_5NO_2$ – H]$^-$; **435** [lyso-PS(18:1) – $C_3H_5NO_2$ + H_2O – H]$^-$; **463** [lyso-PS(22:6) – $C_3H_5NO_2$ – H]$^-$; **481** [lyso-PS(22:6) – $C_3H_5NO_2$ + H_2O – H]$^-$; **745** [M – $C_3H_5NO_2$ – H]$^-$
PS 40:6	C18:0/C22:6	$[M - H]^-$	834	**283** [C18:0 – H]$^-$; **327** [C22:6 – H]$^-$; **419** [lyso-PS(18:0) – $C_3H_5NO_2$ – H]$^-$; **437** [lyso-PS(18:0) – $C_3H_5NO_2$ + H_2O – H]$^-$; **463** [lyso-PS(22:6) – $C_3H_5NO_2$ – H]$^-$; **481** [lyso-PS(22:6) – $C_3H_5NO_2$ + H_2O – H]$^-$; **747** [M – $C_3H_5NO_2$ – H]$^-$
PS 40:5	C18:0/C22:5	$[M - H]^-$	836	**283** [C18:0 – H]$^-$; **329** [C22:5 – H]$^-$; **419** [lyso-PS(18:0) – $C_3H_5NO_2$ – H]$^-$; **437** [lyso-PS(18:0) – $C_3H_5NO_2$ + H_2O – H]$^-$; **465** [lyso-PS(22:5) – $C_3H_5NO_2$ – H]$^-$; **483** [lyso-PS(22:5) – $C_3H_5NO_2$ + H_2O – H]$^-$; **749** [M – $C_3H_5NO_2$ – H]$^-$

PS 40:3	C18:1/C22:2	[M − H]$^-$	840	**281** [C18:1 – H]$^-$; **335** [C22:2 – H]$^-$; **417** [lyso-PS(18:1) – $C_3H_5NO_2$ – H]$^-$; **435** [lyso-PS(18:1) – $C_3H_5NO_2$ + H_2O – H]$^-$; **471** [lyso-PS(22:2) – $C_3H_5NO_2$ + H_2O – H]$^-$; **489** [lyso-PS(22:2) – $C_3H_5NO_2$ + H_2O – H]$^-$; **753** [M – $C_3H_5NO_2$ – H]$^-$
PS 40:2	C18:1/C22:1	[M − H]$^-$	842	**281** [C18:1 – H]$^-$; **337** [C22:1 – H]$^-$; **417** [lyso-PS(18:1) – $C_3H_5NO_2$ – H]$^-$; **435** [lyso-PS(18:1) – $C_3H_5NO_2$ + H_2O – H]$^-$; **473** [lyso-PS(22:1) – $C_3H_5NO_2$ – H]$^-$; **491** [lyso-PS(22:1) – $C_3H_5NO_2$ + H_2O – H]$^-$; **755** [M – $C_3H_5NO_2$ – H]$^-$
PS 40:1	C18:1/C22:0	[M − H]$^-$	844	**281** [C18:1 – H]$^-$; **339** [C22:0 – H]$^-$; **417** [lyso-PS(18:1) – $C_3H_5NO_2$ – H]$^-$; **435** [lyso-PS(18:1) – $C_3H_5NO_2$ + H_2O – H]$^-$; **475** [lyso-PS(22:0) – $C_3H_5NO_2$ – H]$^-$; **493** [lyso-PS(22:0) – $C_3H_5NO_2$ + H_2O – H]$^-$; **757** [M – $C_3H_5NO_2$ – H]$^-$
PS 40:0	C18:0/C22:0	[M − H]$^-$	846	**283** [C18:0 – H]$^-$; **339** [C22:0 – H]$^-$; **419** [lyso-PS(18:0) – $C_3H_5NO_2$ – H]$^-$; **437** [lyso-PS(18:0) – $C_3H_5NO_2$ + H_2O – H]$^-$; **475** [lyso-PS(22:0) – $C_3H_5NO_2$ – H]$^-$; **493** [lyso-PS(22:0) – $C_3H_5NO_2$ + H_2O – H]$^-$; **759** [M – $C_3H_5NO_2$ – H]$^-$
PS 42:3	C20:3/C22:0	[M − H]$^-$	868	**305** [C20:3 – H]$^-$; **339** [C22:0 – H]$^-$; **441** [lyso-PS(20:3) – $C_3H_5NO_2$ – H]$^-$; **459** [lyso-PS(20:3) – $C_3H_5NO_2$ + H_2O – H]$^-$; **475** [lyso-PS(22:0) – $C_3H_5NO_2$ – H]$^-$; **493** [lyso-PS(22:0) – $C_3H_5NO_2$ + H_2O – H]$^-$; **781** [M – $C_3H_5NO_2$ – H]$^-$
PS 42:2	C18:1/C24:1	[M − H]$^-$	870	**281** [C18:1 – H]$^-$; **365** [C24:1 – H]$^-$; **417** [lyso-PS(18:1) – $C_3H_5NO_2$ – H]$^-$; **435** [lyso-PS(18:1) – $C_3H_5NO_2$ + H_2O – H]$^-$; **501** [lyso-PS(24:1) – $C_3H_5NO_2$ – H]$^-$; **519** [lyso-PS(24:1) – $C_3H_5NO_2$ + H_2O – H]$^-$; **783** [M – $C_3H_5NO_2$ – H]$^-$
PS 42:1	C18:1/C24:0	[M − H]$^-$	872	**281** [C18:1 – H]$^-$; **367** [C24:0 – H]$^-$; **417** [lyso-PS(18:1) – $C_3H_5NO_2$ – H]$^-$; **435** [lyso-PS(18:1) – $C_3H_5NO_2$ + H_2O – H]$^-$; **503** [lyso-PS(24:0) – $C_3H_5NO_2$ – H]$^-$; **521** [lyso-PS(24:0) – $C_3H_5NO_2$ + H_2O – H]$^-$; **785** [M – $C_3H_5NO_2$ – H]$^-$
Phosphatidylinositol (PI)				
PI 32:1	C16:0/C16:1	[M − H]$^-$	807	**241** [$C_6H_{10}O_8P$]$^-$; 253 [C16:1 – H]$^-$; **255** [C16:0 – H]$^-$; **389** [lyso-PI(16:1) – $C_6H_{12}O_6$ – H]$^-$; **391** [lyso-PI(16:0) – $C_6H_{12}O_6$ – H]$^-$; **551** [lyso-PI(16:1) – H_2O – H]$^-$; **553** [lyso-PI(16:0) – H_2O – H]$^-$; **569** [lyso-PI(16:1) – H]$^-$; **571** [lyso-PI(16:0) – H]$^-$;
PI 34:2	C16:1/C18:1	[M − H]$^-$	833	**241** [$C_6H_{10}O_8P$]$^-$; **253** [C16:1 – H]$^-$; **281** [C18:1 – H]$^-$; **297** [$C_9H_{14}O_9P$]$^-$; **315** [$C_9H_{16}O_{10}P$]$^-$; **389** [lyso-PI(16:1) – $C_6H_{12}O_6$ – H]$^-$; **417** [lyso-PI(18:1) – $C_6H_{12}O_6$ – H]$^-$; **551** [lyso-PI(16:1) – H_2O – H]$^-$; **569** [lyso-PI(16:1) – H]$^-$; **579** [lyso-PI(18:1) – H_2O – H]$^-$; **597** [lyso-PI(18:1) – H]$^-$
PI 34:1	C16:0/C18:1	[M − H]$^-$	835	**241** [$C_6H_{10}O_8P$]$^-$; **255** [C16:0 – H]$^-$; **281** [C18:1 – H]$^-$; **297** [$C_9H_{14}O_9P$]$^-$; **315** [$C_9H_{16}O_{10}P$]$^-$; **391** [lyso-PI(16:0) – $C_6H_{12}O_6$ – H]$^-$; **417** [lyso-PI(18:1) – $C_6H_{12}O_6$ – H]$^-$; **553** [lyso-PI(16:0) – H_2O – H]$^-$; **571** [lyso-PI(16:0) – H]$^-$; **579** [lyso-PI(18:1) – H_2O – H]$^-$; **597** [lyso-PI(18:1) – H]$^-$

PI 34:0	C16:0/C18:0	$[M-H]^-$	837	**241** [$C_6H_{10}O_8P$]$^-$; **255** [C16:0 – H]$^-$; **283** [C18:0 – H]$^-$; **297** [$C_9H_{14}O_9P$]$^-$; **315** [$C_9H_{16}O_{10}P$]$^-$; **391** [lyso-PI(16:0) – $C_6H_{12}O_6$ – H]$^-$; **419** [lyso-PI(18:0) – $C_6H_{12}O_6$ – H]$^-$; **553** [lyso-PI(16:0) – H_2O – H]$^-$; **571** [lyso-PI(16:0) – H]$^-$; **581** [lyso-PI(18:0) – H_2O – H]$^-$; **599** [lyso-PI(18:0) – H]$^-$
PI 36:4	C16:0/C20:4	$[M-H]^-$	857	**241** [$C_6H_{10}O_8P$]$^-$; 255 [C16:0 – H]$^-$; **303** [C20:4 – H]$^-$; 391 [lyso-PI(16:0) – $C_6H_{12}O_6$ – H]$^-$; 439 [lyso-PI(20:4) – $C_6H_{12}O_6$ – H]$^-$; **553** [lyso-PI(16:0) – H_2O – H]$^-$; **571** [lyso-PI(16:0) – H]$^-$; **601** [lyso-PI(20:4) – H_2O – H]$^-$; **619** [lyso-PI(20:4) – H]$^-$
PI 36:3	C18:1/C18:2	$[M-H]^-$	859	**241** [$C_6H_{10}O_8P$]$^-$; **279** [C18:2 – H]$^-$; **281** [C18:1 – H]$^-$; **297** [$C_9H_{14}O_9P$]$^-$; **315** [$C_9H_{16}O_{10}P$]$^-$; **415** [lyso-PI(18:2) – $C_6H_{12}O_6$ – H]$^-$; **417** [lyso-PI(18:1) – $C_6H_{12}O_6$ – H]$^-$; **577** [lyso-PI(18:2) – H_2O – H]$^-$; **579** [lyso-PI(18:1) – H_2O – H]$^-$; **595** [lyso-PI(18:2) – H]$^-$; **597** [lyso-PI(18:1) – H]$^-$
PI 36:2	C18:1/C18:1	$[M-H]^-$	861	**241** [$C_6H_{10}O_8P$]$^-$; **281** [C18:1 – H]$^-$; **297** [$C_9H_{14}O_9P$]$^-$; **315** [$C_9H_{16}O_{10}P$]$^-$; **417** [lyso-PI(18:1) – $C_6H_{12}O_6$ – H]$^-$; **579** [lyso-PI(18:1) – H_2O – H]$^-$; **597** [lyso-PI(18:1) – H]$^-$
PI 36:1	C18:0/C18:1	$[M-H]^-$	863	**241** [$C_6H_{10}O_8P$]$^-$; **281** [C18:1 – H]$^-$; **283** [C18:0 – H]$^-$; **297** [$C_9H_{14}O_9P$]$^-$; **315** [$C_9H_{16}O_{10}P$]$^-$; **417** [lyso-PI(18:1) – $C_6H_{12}O_6$ – H]$^-$; **419** [lyso-PI(18:0) – $C_6H_{12}O_6$ – H]$^-$; **579** [lyso-PI(18:1) – H_2O – H]$^-$; **581** [lyso-PI(18:0) – H_2O – H]$^-$; **597** [lyso-PI(18:1) – H]$^-$; **599** [lyso-PI(18:0) – H]$^-$
PI 36:0	C18:0/C18:0	$[M-H]^-$	865	**241** [$C_6H_{10}O_8P$]$^-$; **283** [C18:0 – H]$^-$; **297** [$C_9H_{14}O_9P$]$^-$; **315** [$C_9H_{16}O_{10}P$]$^-$; **419** [lyso-PI(18:0) – $C_6H_{12}O_6$ – H]$^-$; **581** [lyso-PI(18:0) – H_2O – H]$^-$; **599** [lyso-PI(18:0) – H]$^-$
PI 38:5	C18:1/C20:4	$[M-H]^-$	883	**241** [$C_6H_{10}O_8P$]$^-$; **281** [C18:1 – H]$^-$; **297** [$C_9H_{14}O_9P$]$^-$; **303** [C20:4 – H]$^-$; **315** [$C_9H_{16}O_{10}P$]$^-$; **417** [lyso-PI(18:1) – $C_6H_{12}O_6$ – H]$^-$; **439** [lyso-PI(20:4) – $C_6H_{12}O_6$ – H]$^-$; **579** [lyso-PI(18:1) – H_2O – H]$^-$; **597** [lyso-PI(18:1) – H]$^-$; **601** [lyso-PI(20:4) – H_2O – H]$^-$; **619** [lyso-PI(20:4) – H]$^-$
PI 38:4	C18:0/C20:4	$[M-H]^-$	885	**241** [$C_6H_{10}O_8P$]$^-$; **283** [C18:0 – H]$^-$; **297** [$C_9H_{14}O_9P$]$^-$; **303** [C20:4 – H]$^-$; **315** [$C_9H_{16}O_{10}P$]$^-$; **419** [lyso-PI(18:0) – $C_6H_{12}O_6$ – H]$^-$; **439** [lyso-PI(20:4) – $C_6H_{12}O_6$ – H]$^-$; **581** [lyso-PI(18:0) – H_2O – H]$^-$; **599** [lyso-PI(18:0) – H]$^-$; **601** [lyso-PI(20:4) – H_2O – H]$^-$; **619** [lyso-PI(20:4) – H]$^-$
PI 38:3	C18:0/C20:3	$[M-H]^-$	887	**241** [$C_6H_{10}O_8P$]$^-$; **283** [C18:0 – H]$^-$; **297** [$C_9H_{14}O_9P$]$^-$; **305** [C20:3 – H]$^-$; **315** [$C_9H_{16}O_{10}P$]$^-$; **419** [lyso-PI(18:0) – $C_6H_{12}O_6$ – H]$^-$; **441** [lyso-PI(20:3) – $C_6H_{12}O_6$ – H]$^-$; **581** [lyso-PI(18:0) – H_2O – H]$^-$; **599** [lyso-PI(18:0) – H]$^-$; **603** [lyso-PI(20:3) – H_2O – H]$^-$; **621** [lyso-PI(20:3) – H]$^-$
PI 38:2	C18:0/C20:2	$[M-H]^-$	889	**241** [$C_6H_{10}O_8P$]$^-$; **283** [C18:0 – H]$^-$; **297** [$C_9H_{14}O_9P$]$^-$; **307** [C20:2 – H]$^-$; **315** [$C_9H_{16}O_{10}P$]$^-$; **419** [lyso-PI(18:0) – $C_6H_{12}O_6$ – H]$^-$; **443** [lyso-PI(20:2) – $C_6H_{12}O_6$ – H]$^-$; **581** [lyso-PI(18:0) – H_2O – H]$^-$; **599** [lyso-PI(18:0) – H]$^-$; **605** [lyso-PI(20:2) – H_2O – H]$^-$; **623** [lyso-PI(20:2) – H]$^-$

Supplementary Table S4. Fold-changes and *p*-values of lipid species identified from melanoma cells (A375 and A2058) and human epidermal melanocytes (HEMn-LP) by nanoESI-MS. The log2(FC) presents the $\log_2$ ratio of peak intensity of identified lipid species in COR-treated group versus untreated groups. Positive values of log2(FC) indicate an increase in the COR-treated groups, whereas negative values indicate a decrease. The *p*-values were obtained by the Student's *t*-test. ns, not significant difference ($p > 0.05$); ↑, significantly increased level compared with control ($p < 0.05$); ↓, significantly decreased level compared with control ($p < 0.05$).

Lipid molecular species	m/z	HEMn-LP				A375				A2058			
		1.0 µM COR		2-DG + 0.01 µM COR		1.0 µM COR		2-DG + 0.01 µM COR		1.0 µM COR		2-DG + 0.01 µM COR	
		p	log2(FC)	*p*	log2(FC)	*p*	log2(FC)	*p*	log2(FC)	*p*	log2(FC)	*p*	log2(FC)
**Positive ion mode**													
**Phosphatidylcholine (PC)**													
PC (14:0/16:0)	706	0.463	-0.08 (ns)	0.049	-0.28 (↓)	0.001	0.49 (↑)	0.004	0.28 (↑)	0.005	-0.55 (↓)	0.002	-0.45 (↓)
PC (16:0/16:1)	732	0.509	-0.06 (ns)	0.093	-0.19 (ns)	0.000	0.52 (↑)	0.015	0.16 (↑)	0.001	-0.54 (↓)	0.002	-0.49 (↓)
PC (16:0/16:0)	734	0.384	-0.05 (ns)	0.070	-0.17 (ns)	0.001	0.52 (↑)	0.001	0.40 (↑)	0.137	-0.15 (ns)	0.238	-0.09 (ns)
PC (P-16:0/16:0)	740	0.914	-0.02 (ns)	0.652	-0.17 (ns)	0.098	0.26 (ns)	0.226	-0.11 (ns)	0.394	-0.28 (ns)	0.141	-0.55 (ns)
PC (16:1/18:1)	758	0.197	-0.12 (ns)	0.080	-0.18 (ns)	0.000	0.60 (↑)	0.000	0.36 (↑)	0.050	-0.27 (ns)	0.083	-0.16 (ns)
PC (16:0/18:1)	760	0.275	-0.06 (ns)	0.060	-0.15 (ns)	0.000	0.56 (↑)	0.002	0.33 (↑)	0.007	-0.33 (↓)	0.044	-0.18 (↓)
PC (16:0/18:0)	762	0.035	-0.12 (↓)	1.000	3.0E-05 (ns)	0.000	0.57 (↑)	0.001	0.51 (↑)	0.271	0.24 (ns)	0.008	0.36 (↑)
PC (P-16:0/18:0)	768	0.455	-0.16 (ns)	0.229	-0.29 (ns)	0.000	0.50 (↑)	0.000	0.45 (↑)	0.488	0.07 (ns)	0.037	0.21 (↑)
PC (18:1/18:1)	786	0.000	-0.32 (↓)	0.112	0.13 (ns)	0.000	0.69 (↑)	0.036	0.60 (↑)	0.209	0.34 (ns)	0.002	0.63 (↑)
PC (18:0/18:1)	788	0.000	-0.40 (↓)	0.110	0.08 (ns)	0.001	0.68 (↑)	0.011	0.59 (↑)	0.084	0.62 (ns)	0.002	0.93 (↑)
PC (18:1/18:2)	806	0.056	-0.56 (ns)	0.860	-0.05 (ns)	0.000	0.70 (↑)	0.060	0.51 (ns)	0.059	0.91 (ns)	0.013	0.80 (↑)

**Triacylglycerol (TG)**													
TG (16:0/16:1/18:1)	848	0.789	0.06 (ns)	0.879	0.03 (ns)	0.258	0.33 (ns)	0.022	0.65 (↑)	0.131	0.34 (ns)	0.039	0.12 (↑)
TG (16:0/16:1/18:0)	850	0.548	0.08 (ns)	0.699	-0.06 (ns)	0.008	0.71 (↑)	0.001	1.03 (↑)	0.004	0.45 (↑)	0.215	0.15 (ns)
TG (16:1/18:1/18:1)	874	0.021	0.71 (↑)	0.042	0.60 (↑)	0.055	0.53 (ns)	0.001	1.38 (↑)	0.031	0.91 (↑)	0.630	-0.13 (ns)
TG (16:0/18:1/18:1)	876	0.000	0.86 (↑)	0.000	0.85 (↑)	0.006	0.60 (↑)	0.000	1.24 (↑)	0.165	0.23 (ns)	0.128	-0.23 (ns)
TG (16:0/18:0/18:1)	878	0.00	1.22 (↑)	0.004	0.87 (↑)	0.002	0.69 (↑)	0.000	0.98 (↑)	0.041	0.39 (↑)	0.215	-0.11 (ns)
TG (18:1/18:1/18:1)	902	0.001	0.98 (↑)	0.000	0.98 (↑)	0.140	0.54 (ns)	0.003	1.33 (↑)	0.056	0.59 (ns)	0.370	0.12 (ns)
**Negative ion mode**													
**Ceramide (Cer)**													
Cer (d18:1/16:1)	534	0.008	-0.28 (↓)	0.001	-0.36 (↓)	0.021	0.98 (↑)	0.152	0.23 (ns)	0.252	0.33 (ns)	0.012	0.78 (↑)
Cer (d18:1/16:0)	536	0.010	-0.21 (↓)	0.001	-0.28 (↓)	0.004	0.77 (↑)	0.000	0.85 (↑)	0.031	0.72 (↑)	0.003	0.89 (↑)
Cer (d18:1/18:1)	562	0.002	-0.32 (↓)	0.001	-0.32 (↓)	0.013	0.97 (↑)	0.000	0.67 (↑)	0.014	1.03 (↑)	0.001	1.31 (↑)
Cer (d18:1/18:0)	564	0.001	-0.34 (↓)	0.031	-0.13 (↓)	0.000	0.98 (↑)	0.000	1.20 (↑)	0.006	1.46 (↑)	0.008	1.72 (↑)
Cer (d18:0/18:0)	566	0.001	-0.35 (↓)	0.002	-0.31 (↓)	0.000	0.85 (↑)	0.000	0.98 (↑)	0.055	1.01 (ns)	0.001	1.42 (↑)
Cer (d18:1/20:1)	590	0.428	-0.06 (ns)	0.001	-0.55 (↓)	0.081	0.42 (ns)	0.001	0.55 (↑)	0.431	0.12 (ns)	0.018	0.25 (↑)
Cer (d18:1/20:0)	592	0.673	-0.03 (ns)	0.004	-0.20 (↓)	0.019	0.41 (↑)	0.001	1.14 (↑)	0.007	0.94 (↑)	0.002	0.84 (↑)
Cer (d18:0/20:0)	594	0.000	-0.46 (↓)	0.000	-0.21 (↓)	0.023	0.39 (↑)	0.000	0.68 (↑)	0.003	0.77 (↑)	0.000	0.79 (↑)
Cer (d18:1/22:0)	620	0.037	0.18 (↑)	0.010	-0.14 (↓)	0.024	0.53 (↑)	0.000	1.43 (↑)	0.000	1.29 (↑)	0.000	0.71 (↑)
**Phosphatidylethanolamine (PE)**													
PE (P-16:0/18:1)	700	0.004	0.06 (↑)	0.005	-0.05 (↓)	0.503	-0.06 (ns)	0.000	0.48 (↑)	0.006	-0.43 (↓)	0.000	-0.89 (↓)
PE (16:1/18:1)	714	0.932	-0.01 (ns)	0.127	-0.07 (ns)	0.274	0.09 (ns)	0.001	0.45 (↑)	0.266	-0.14 (ns)	0.001	-0.25 (↓)

PE (16:0/18:1)	716	0.000	-0.10 (↓)	0.000	-0.20 (↓)	0.098	-0.09 (ns)	0.525	-0.01 (ns)	0.662	-0.04 (ns)	0.001	-0.39 (↓)
PE (P-16:0/20:4)	722	0.565	0.02 (ns)	0.329	-0.04 (ns)	0.136	0.09 (ns)	0.002	0.19 (↑)	0.050	0.11 (ns)	0.325	-0.05 (ns)
PE (18:1/18:1)	742	0.073	-0.05 (ns)	0.001	-0.17 (↓)	0.049	-0.10 (↓)	0.068	-0.06 (ns)	0.001	-0.36 (↓)	0.001	-0.51 (↓)
PE (18:0/18:1)	744	0.001	-0.16 (↓)	0.000	-0.32 (↓)	0.212	-0.04 (ns)	0.088	-0.05 (ns)	0.169	-0.13 (ns)	0.011	-0.38 (↓)
PE (P-16:0/22:6)	746	0.000	-0.21 (↓)	0.004	-0.37 (↓)	0.293	-0.06 (ns)	0.009	0.18 (↑)	0.394	0.06 (ns)	0.008	-0.15 (↓)
PE (P-16:0/22:5)	748	0.015	0.03 (↑)	0.648	4.8E-03 (ns)	0.645	-0.02 (ns)	0.212	-0.05 (ns)	0.055	-0.20 (ns)	0.013	-0.27 (↓)
PE (P-18:0/20:4)	750	0.076	0.05 (ns)	0.050	-0.05 (ns)	0.502	0.01 (ns)	0.049	0.06 (↑)	0.197	0.16 (ns)	0.928	0.01 (ns)
PE (18:1/20:4)	764	0.015	-0.10 (↓)	0.006	-0.14 (↓)	0.049	0.30 (↑)	0.000	0.40 (↑)	0.019	0.51 (↑)	0.004	0.57 (↑)
PE (18:0/20:4)	766	0.436	0.03 (ns)	0.105	-0.05 (ns)	0.107	0.12 (ns)	0.001	0.37 (↑)	0.002	0.60 (↑)	0.064	0.40 (ns)
PE (P-18:0/22:5)	776	0.000	-0.43 (↓)	0.000	-0.90 (↓)	0.000	-1.09 (↓)	0.002	0.36 (↑)	0.074	0.13 (ns)	0.000	0.68 (↑)
PE (P-18:0/22:4)	778	0.926	-4.8E-03 (ns)	0.025	-0.19 (↓)	0.162	-0.19 (ns)	0.005	0.45 (↑)	0.131	0.21 (ns)	0.521	0.06 (ns)
PE (18:0/22:5)	792	0.003	-0.16 (↓)	0.002	-0.22 (↓)	0.146	0.20 (ns)	0.001	0.29 (↑)	0.039	0.48 (↑)	0.000	0.58 (↑)
PE (18:0/22:4)	794	0.027	0.14 (↑)	0.065	-0.11 (ns)	0.098	0.15 (ns)	0.000	0.64 (↑)	0.013	0.98 (↑)	0.203	1.08 (ns)
**Phosphatidylglycerol (PG)**													
PG (16:0/18:1)	747	0.711	-0.02 (ns)	0.007	-0.22 (↓)	0.011	-0.27 (↓)	0.008	-0.27 (↓)	0.000	-0.69 (↓)	0.001	-0.62 (↓)
PG (18:1/18:1)	773	0.728	0.03 (ns)	0.000	-0.26 (↓)	0.011	-1.03 (↓)	0.010	-1.67 (↓)	0.000	-2.24 (↓)	0.000	-2.36 (↓)
PG (18:0/18:1)	775	0.972	-1.7E-03 (ns)	0.004	-0.10 (↓)	0.001	-0.34 (↓)	0.000	-0.33 (↓)	0.001	-0.61 (↓)	0.000	-0.73 (↓)
**Phosphatidylserine (PS)**													
PS (14:0/16:0)	706	0.016	-0.26 (↓)	0.000	-0.68 (↓)	0.371	0.05 (ns)	0.798	-0.02 (ns)	0.008	-0.49 (↓)	0.003	-0.68 (↓)
PS (16:1/18:1)	758	0.000	-0.32 (↓)	0.005	-0.27 (↓)	0.018	0.68 (↑)	0.000	0.60 (↑)	0.578	-0.11 (ns)	0.000	0.79 (↑)
PS (16:0/18:1)	760	0.000	-0.39 (↓)	0.003	-0.28 (↓)	0.063	0.62 (ns)	0.000	0.94 (↑)	0.203	0.33 (ns)	0.000	1.23 (↑)
PS (16:0/18:0)	762	0.000	-0.25 (↓)	0.065	-0.09 (ns)	0.068	0.61 (ns)	0.002	1.00 (↑)	0.031	0.84 (↑)	0.000	1.31 (↑)
PS (18:1/18:2)	784	0.000	-0.73 (↓)	0.005	-0.56 (↓)	0.058	1.05 (ns)	0.000	0.64 (↑)	0.143	0.65 (ns)	0.001	2.02 (↑)

PS (18:1/18:1)	786	0.001	-0.30 (↓)	0.004	-0.31 (↓)	0.000	0.94 (↑)	0.006	0.48 (↑)	0.178	-0.23 (ns)	0.000	0.46 (↑)
PS (18:0/18:1)	788	0.002	0.08 (↑)	0.762	3.7E-03 (ns)	0.281	0.14 (ns)	0.000	0.46 (↑)	0.566	-0.06 (ns)	0.000	0.45 (↑)
PS (18:0/18:0)	790	0.107	-0.05 (ns)	0.059	-0.06 (ns)	0.000	0.39 (↑)	0.000	0.28 (↑)	0.394	0.09 (ns)	0.001	0.39 (↑)
PS (18:1/19:0)	802	0.000	-0.26 (↓)	0.000	-0.59 (↓)	0.190	-0.16 (ns)	0.073	-0.15 (ns)	0.719	-0.02 (ns)	0.002	0.26 (↑)
PS (16:0/22:6)	806	0.357	-0.03 (ns)	0.002	-0.27 (↓)	0.087	0.14 (ns)	0.004	0.36 (↑)	0.217	0.18 (ns)	0.077	0.25 (ns)
PS (18:1/20:4)	808	0.609	0.01 (ns)	0.049	-0.13 (↓)	0.165	0.30 (ns)	0.000	0.82 (↑)	0.063	0.28 (ns)	0.002	0.89 (↑)
PS (18:0/20:4)	810	0.000	0.45 (↑)	0.001	0.37 (↑)	0.579	0.03 (ns)	0.000	0.72 (↑)	0.000	0.40 (↑)	0.000	0.72 (↑)
PS (18:0/20:3)	812	0.001	0.28 (↑)	0.008	0.21 (↑)	0.013	-0.23 (↓)	0.007	0.18 (↑)	0.000	0.55 (↑)	0.003	0.36 (↑)
PS (18:1/20:1)	814	0.005	0.08 (↑)	0.588	0.01 (ns)	0.007	0.35 (↑)	0.070	0.09 (ns)	0.002	-0.44 (↓)	0.012	-0.19 (↓)
PS (18:1/20:0)	816	0.004	-0.10 (↓)	0.188	-0.03 (ns)	0.002	0.45 (↑)	0.000	0.29 (↑)	0.371	0.11 (ns)	0.000	0.45 (↑)
PS (18:1/22:6)	832	0.239	-0.04 (ns)	0.642	-0.01 (ns)	0.193	0.30 (ns)	0.000	0.43 (↑)	0.105	0.37 (ns)	0.002	0.32 (↑)
PS (18:0/22:6)	834	0.009	0.14 (↑)	0.028	0.06 (↑)	0.237	0.08 (ns)	0.001	0.17 (↑)	0.398	-0.05 (ns)	0.104	0.22 (ns)
PS (18:0/22:5)	836	0.002	0.18 (↑)	0.005	0.11 (↑)	0.082	-0.10 (ns)	0.023	0.10 (↑)	0.007	-0.34 (↓)	0.236	0.17 (ns)
PS (18:1/22:2)	840	0.000	0.19 (↑)	0.500	0.02 (ns)	0.021	0.48 (↑)	0.000	0.85 (↑)	0.003	0.52 (↑)	0.004	0.71 (↑)
PS (18:1/22:1)	842	0.999	3.7E-05 (ns)	0.862	-3.1E-03 (ns)	0.051	0.31 (ns)	0.001	0.39 (↑)	0.081	0.11 (ns)	0.010	0.36 (↑)
PS (18:1/22:0)	844	0.015	-0.14 (↓)	0.040	-0.09 (↓)	0.002	0.46 (↑)	0.001	0.32 (↑)	0.132	0.15 (ns)	0.007	0.33 (↑)
PS (18:0/22:0)	846	0.004	-0.19 (↓)	0.000	-0.26 (↓)	0.001	0.40 (↑)	0.000	0.40 (↑)	0.003	0.62 (↑)	0.000	0.66 (↑)
PS (20:3/22:0)	868	0.241	-0.04 (ns)	0.063	-0.06 (ns)	0.001	0.45 (↑)	0.000	1.13 (↑)	0.002	0.27 (↑)	0.009	0.28 (↑)
PS (18:1/24:1)	870	0.344	-0.01 (ns)	0.008	-0.10 (↓)	0.006	0.37 (↑)	0.000	0.47 (↑)	0.007	-0.25 (↓)	0.425	0.88 (ns)
PS (18:1/24:0)	872	0.052	-0.06 (ns)	0.001	-0.24 (↓)	0.961	0.01 (ns)	0.100	0.13 (ns)	0.348	0.11 (ns)	0.163	0.14 (ns)
**Phosphatidylinositol (PI)**													
PI (16:0/16:1)	807	0.001	-0.25 (↓)	0.000	-0.29 (↓)	0.073	0.33 (ns)	0.001	0.55 (↑)	0.426	0.25 (ns)	0.007	0.52 (↑)
PI (16:1/18:1)	833	0.901	4.8E-03 (ns)	0.162	-0.05 (ns)	0.047	0.30 (↑)	0.007	0.25 (↑)	0.012	-1.00 (↓)	0.004	-0.41 (↓)
PI (16:0/18:1)	835	0.474	0.06 (ns)	0.784	-0.01 (ns)	0.025	-0.14 (↓)	0.007	-0.15 (↓)	0.000	-1.31 (↓)	0.004	-0.41 (↓)

PI (16:0/18:0)	837	0.000	0.29 (↑)	0.008	0.17 (↑)	0.023	-0.21 (↓)	0.591	-4.9E-03 (ns)	0.006	-0.25 (↓)	0.574	0.25 (ns)
PI (16:0/20:4)	857	0.843	-0.01 (ns)	0.058	0.06 (ns)	0.119	0.19 (ns)	0.012	0.24 (↑)	0.010	0.45 (↑)	0.001	0.94 (↑)
PI (18:1/18:2)	859	0.306	-0.03 (ns)	0.284	0.05 (ns)	0.081	0.26 (ns)	0.013	0.49 (↑)	0.484	0.12 (ns)	0.001	0.62 (↑)
PI (18:1/18:1)	861	0.534	0.03 (ns)	0.026	-0.06 (↓)	0.026	-0.18 (↓)	0.001	-0.26 (↓)	0.000	-1.99 (↓)	0.000	-0.91 (↓)
PI (18:0/18:1)	863	0.351	0.05 (ns)	0.446	-0.03 (ns)	0.001	-0.31 (↓)	0.001	-0.61 (↓)	0.000	-1.96 (↓)	0.000	-0.83 (↓)
PI (18:0/18:0)	865	0.666	0.04 (ns)	0.108	0.15 (ns)	0.015	-0.30 (↓)	0.105	-0.19 (ns)	0.001	-0.55 (↓)	0.203	-0.21 (ns)
PI (18:1/20:4)	883	0.092	0.08 (ns)	0.954	1.4E-03 (ns)	0.629	-0.02 (ns)	0.000	0.23 (↑)	0.000	-0.80 (↓)	0.003	0.26 (↑)
PI (18:0/20:4)	885	0.264	0.05 (ns)	0.189	-0.04 (ns)	0.398	0.05 (ns)	0.000	0.29 (↑)	0.008	-0.19 (↓)	0.000	0.83 (↑)
PI (18:0/20:3)	887	0.457	0.04 (ns)	0.042	-0.08 (↓)	0.079	-0.11 (ns)	0.130	-0.07 (ns)	0.000	-0.78 (↓)	0.002	0.32 (↑)
PI (18:0/20:2)	889	0.268	0.06 (ns)	0.358	-0.04 (ns)	0.014	-0.31 (↓)	0.002	-0.53 (↓)	0.000	-1.87 (↓)	0.000	-1.22 (↓)

Supplementary Table S5. Parameters of OPLS-DA models derived from metabolite and lipid profiling data sets of melanoma cells.

	**A375**		**A2058**	
	**COR vs Control**	**2-DG + COR vs Control**	**COR vs Control**	**2-DG + COR vs Control**
R^2Y	0.985	0.994	0.983	0.995
Q^2Y	0.975	0.992	0.972	0.992
p	3.86E-03	7.56E-04	4.58E-03	7.19E-04

Supplementary Table S6. The metabolites and lipids which were strongly contributed to separation between COR-treated and control groups in the two melanoma cell lines (A375 and A2058). Each of the 10 compounds was selected at the lower left and the upper right region of the S-plots, corresponding to decreased and increased levels after COR and COR + 2-DG treatment, respectively. Note: VIP indicates variable influence on projection value from the orthogonal partial least-squares discriminant analysis (OPLS-DA); p(corr) indicates correlation value from the S-plot.

No.	A375 (COR vs Control)			A375 (COR + 2-DG vs Control)			A2058 (COR vs Control)			A2058 (COR + 2-DG vs Control)		
	**Compound**	**VIP**	**p(corr)**	**Compound**	**VIP**	**p(corr)**	**Compound**	**VIP**	**p(corr)**	**Compound**	**VIP**	**p(corr)**
**At the lower left region of the S-plot**												
1	Glutamic acid	3.894	-0.983	Glutamic acid	3.848	-0.990	Lactic acid	3.604	-0.974	Lactic acid	2.955	-0.960
2	Myo-Inositol	2.932	-0.824	Lactic acid	2.092	-0.998	Glutamic acid	2.498	-0.979	Glutamic acid	2.689	-0.991
3	Aspartic acid	1.785	-0.988	Aspartic acid	1.882	-0.995	PI 18:1/18:1	2.489	-0.998	PI 18:1/18:1	1.675	-0.995
4	Lactic acid	1.763	-0.990	Hypoxanthine	1.278	-0.997	PI 18:0/18:1	2.370	-0.998	PI 18:0/18:1	1.550	-0.995
5	plasmenyl-PE 18:0/22:5	1.429	-0.988	PI 18:0/18:1	1.250	-0.998	PC 16:0/18:1	2.348	-0.935	PG 18:1/18:1	1.504	-0.996
6	Glycine	1.233	-0.983	Glucose	0.977	-0.999	PI 16:0/18:1	2.016	-0.997	PC 16:0/16:1	1.456	-0.963
7	Hypoxanthine	1.065	-0.990	PI 18:1/18:1	0.738	-0.970	PC 16:0/16:1	1.792	-0.982	PC 16:0/18:1	1.419	-0.823
8	PI 18:0/18:1	0.998	-0.975	Malic acid	0.695	-0.976	PG 18:1/18:1	1.743	-0.996	PI 16:0/18:1	1.078	-0.946
9	Pyroglutamic acid	0.822	-0.936	PG 18:1/18:1	0.694	-0.980	PI 16:1/18:1	1.163	-0.972	Valine	0.896	-0.989
10	Threonine	0.737	-0.987	Uracil	0.678	-1.000	PI 18:0/20:2	1.160	-0.996	PI 18:0/20:2	0.879	-0.994
**At the upper right region of the S-plot**												
11	Cer d18:1/18:0	3.415	0.991	Cer d18:1/18:0	3.698	0.998	Cer d18:1/18:0	3.998	0.936	Cer d18:1/18:0	3.994	0.980

12	PC 18:1/18:1	2.605	0.990	PS 16:1/18:0	2.623	0.998	Cer d18:0/18:0	1.306	0.896	PC 18:1/18:1	3.041	0.963
13	PS 18:1/18:1	2.494	0.984	PS 18:0/18:1	2.162	0.998	Cer d18:1/18:1	1.301	0.905	PC 18:0/18:1	2.777	0.964
14	PC 16:0/18:1	2.449	0.985	PC 18:1/18:1	2.123	0.913	Cer d18:1/16:0	1.204	0.854	PS 16:1/18:0	2.491	0.998
15	PS 16:1/18:0	2.031	0.886	PC 16:0/18:1	1.651	0.963	PS 16:0/18:0	1.152	0.849	PS 18:0/18:1	1.988	0.999
16	PC 18:0/18:1	1.992	0.981	PC 18:0/18:1	1.636	0.912	Cer d18:0/20:0	1.011	0.954	PI 18:0/20:4	1.626	0.996
17	Cer d18:1/18:1	1.717	0.973	Cer d18:1/16:0	1.566	0.997	Cer d18:1/20:0	1.004	0.932	Cer d18:0/18:0	1.491	0.972
18	Cer d18:1/16:0	1.541	0.950	PS 18:1/18:1	1.530	0.988	PS 18:0/22:0	0.985	0.957	PS 16:0/18:0	1.455	0.998
19	Cer d18:0/18:0	1.443	0.991	Cer d18:0/18:0	1.488	0.999	PE 18:0/22:5	0.922	0.838	Cer d18:1/18:1	1.378	0.978
20	PC 16:0/16:1	1.391	0.981	PS 16:0/18:0	1.421	0.995	PE 18:0/22:4	0.864	0.905	Cer d18:1/16:0	1.246	0.955

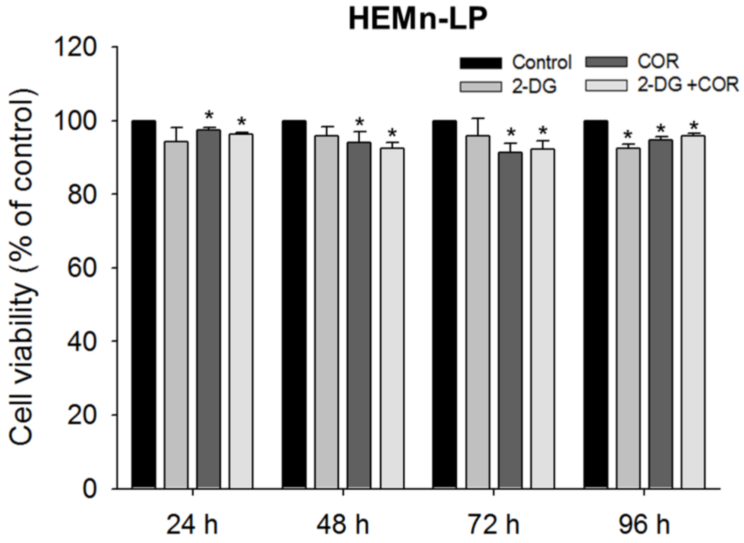

Supplementary Figure S1. Cytotoxicity effect of 0.01 μM COR and 0.5 mM 2-DG on normal melanocytes. The cells were treated for 24, 48, 72 and 96 h with 2-DG or COR alone and with the combination of 2-DG with COR, and cell viability was measured by the MTT assay. The bars indicate mean values, and the error bars indicate the standard deviation of triplicate experiments. The asterisk (*) denotes significant difference compared with the control group, as determined using the Student's *t*-test ($p < 0.05$).

Compound #1

Aspartic acid (3TMS derivative)
Retention time (min): 17.37
Fragmentation ion (m/z): 100, 202, 218, **232**
Molecular Formula: $C_{13}H_{31}NO_4Si_3$

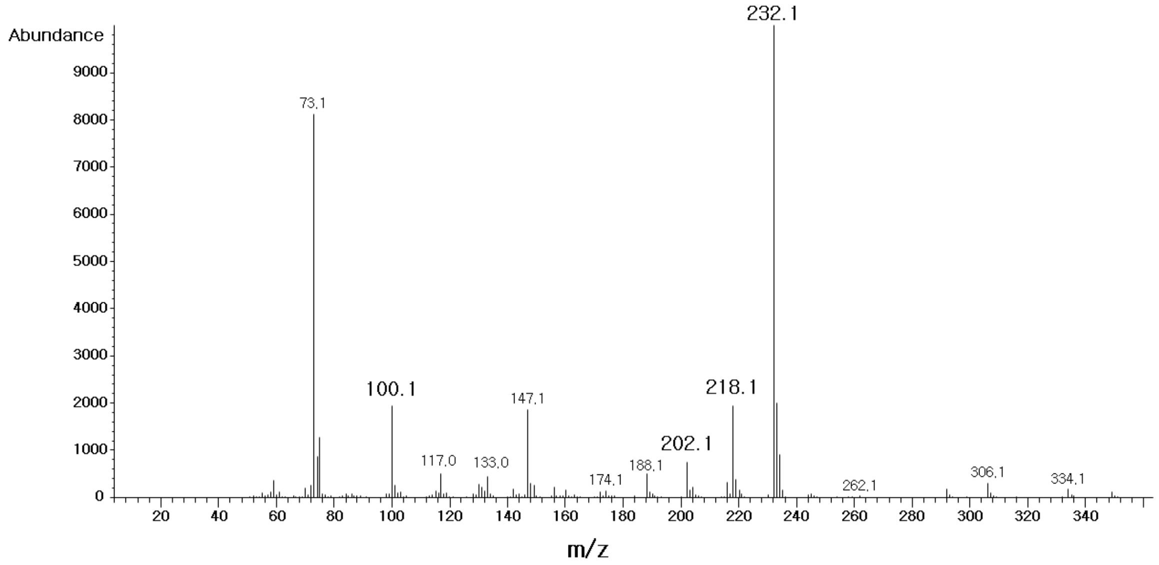

Compound #2

Glutamic acid (3TMS derivative)
Retention time (min): 19.72
Fragmentation ion (m/z): 128, 156, **246**, 348
Molecular Formula: $C_{14}H_{33}NO_4Si_3$

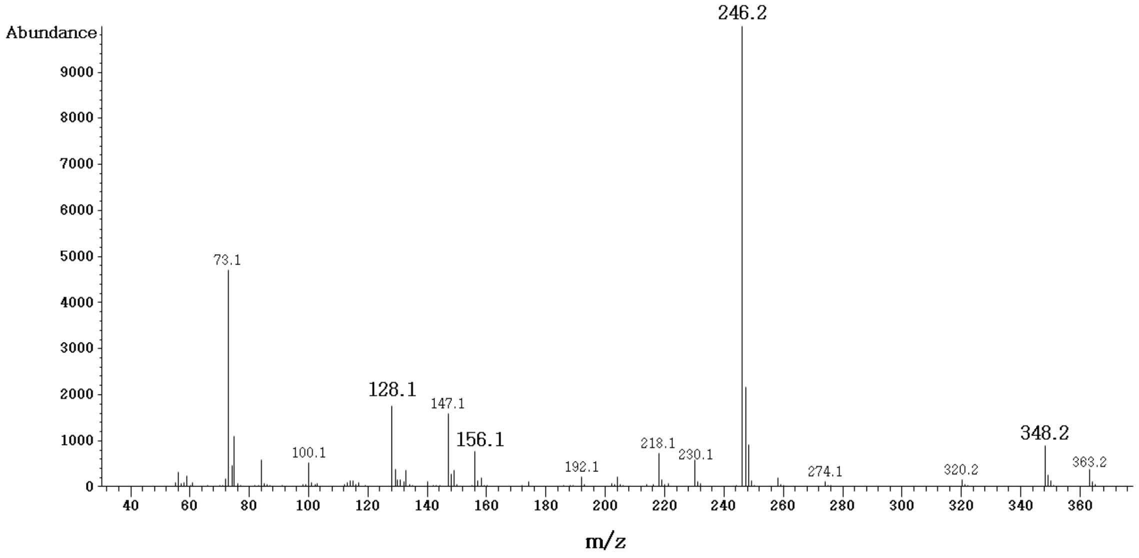

Compound #3

Lactic acid (2TMS derivative)

Retention time (min): 6.02
Fragmentation ion (m/z): **117,** 133, 191, 219
Molecular Formula: $C_9H_{22}O_3Si_2$

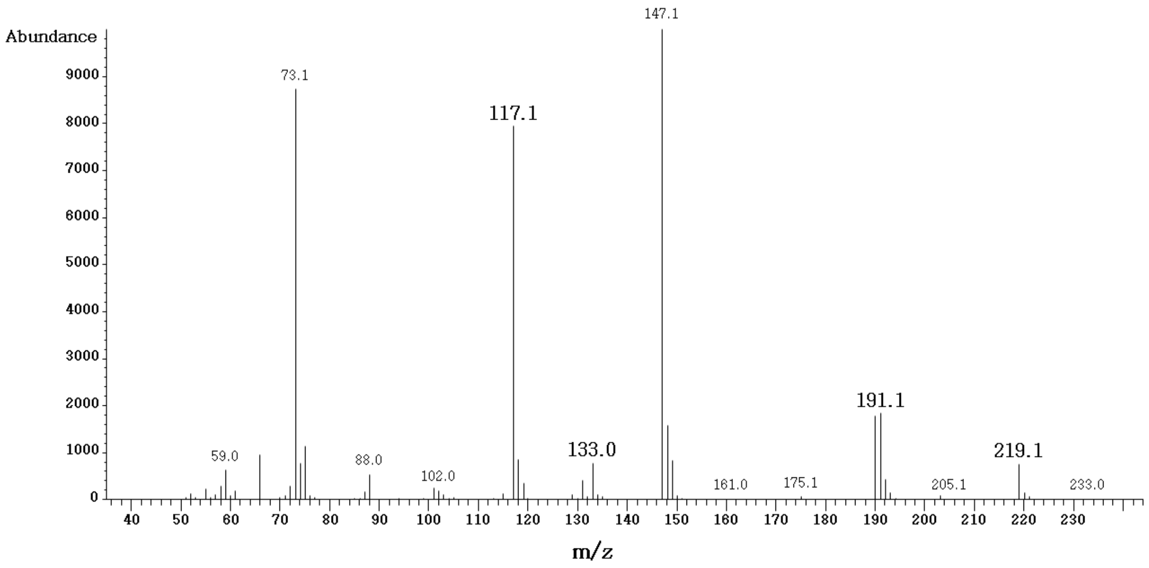

Compound #4

Hypoxanthine (2TMS derivative)

Retention time (min): 23.71
Fragmentation ion (m/z): 193, 206, **265**, 280
Molecular Formula: $C_{11}H_{20}N_4OSi_2$

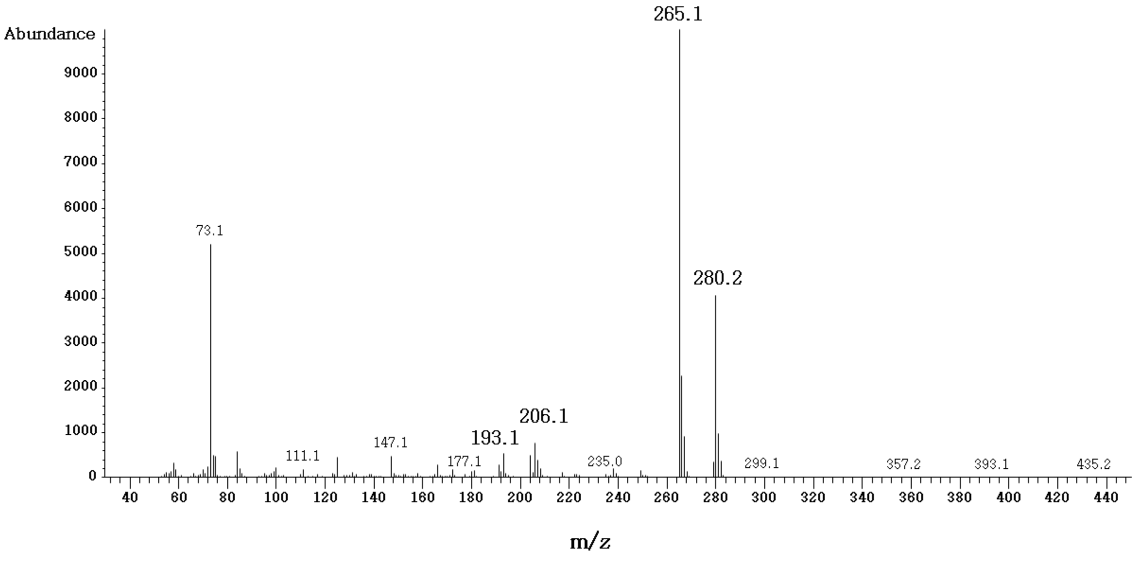

Compound #5

PC (18:1/18:1) [M + H] $^+$, *m/z* 786

• $C_5H_{14}NO_4P$: phosphorylcholine

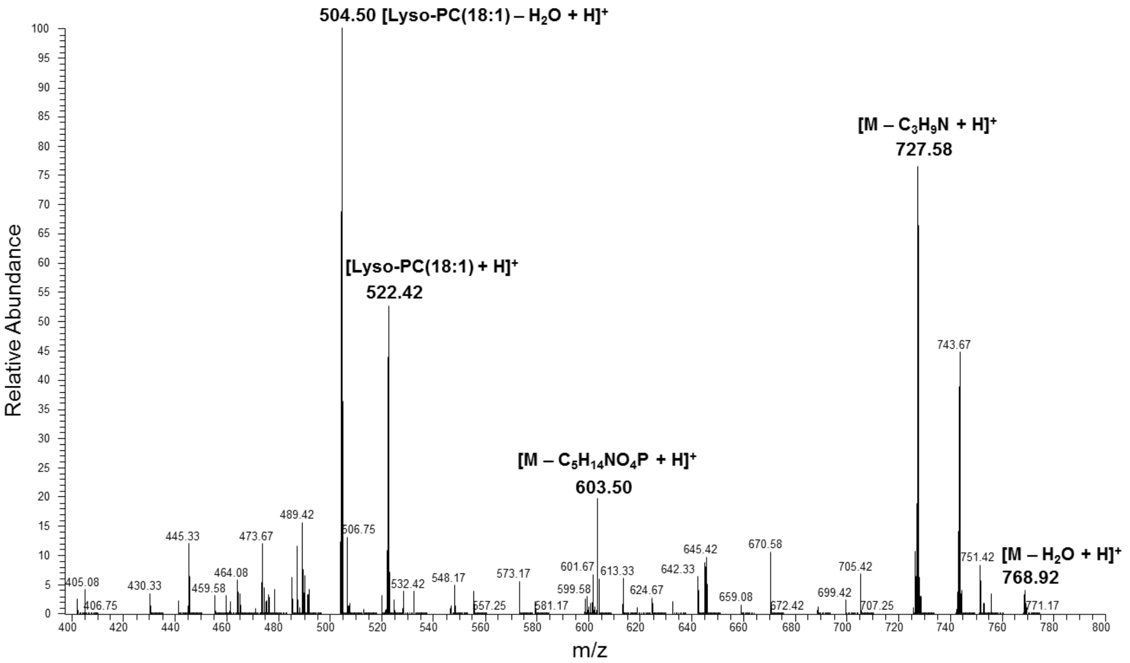

Compound #6

PC (18:0/18:1) [M + H] $^+$, *m/z* 788

• $C_5H_{14}NO_4P$: phosphorylcholine

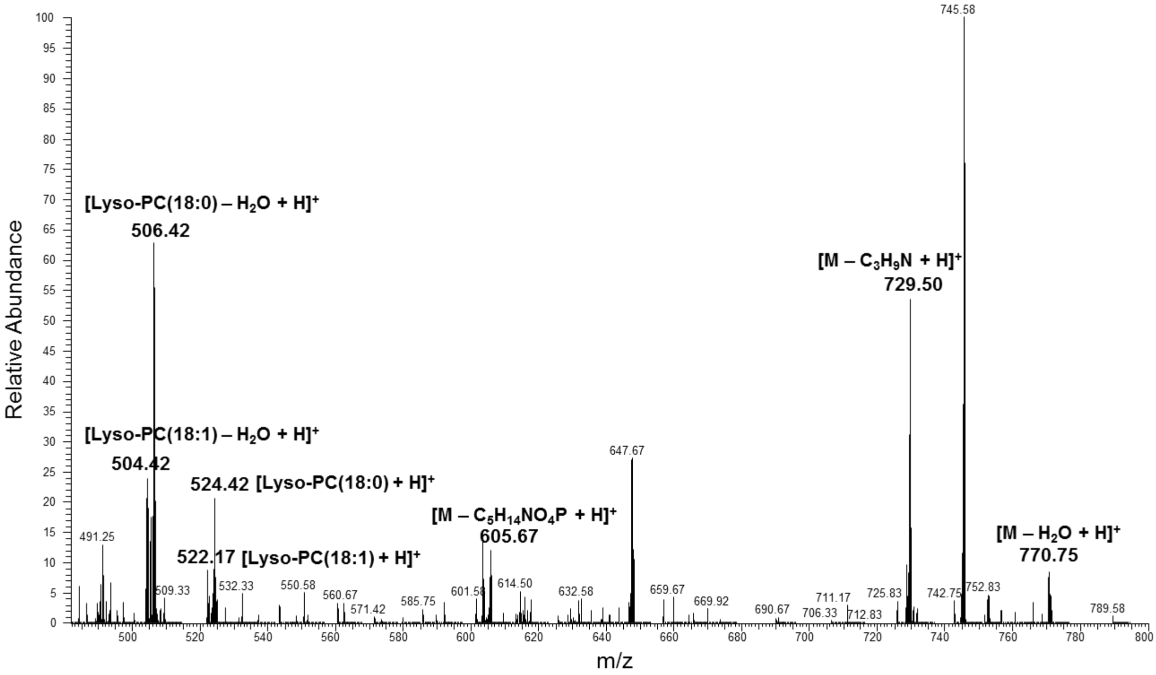

Compound #7

Cer (d18:1/16:0) [M - H]$^-$, *m/z* 536

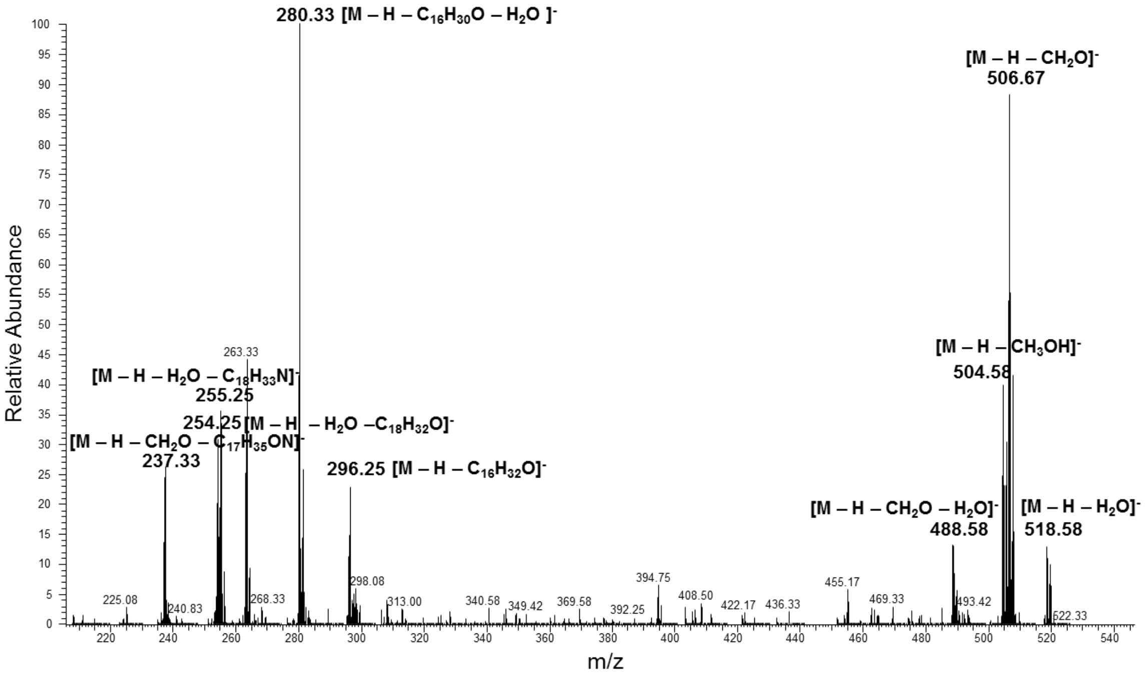

Compound #8

Cer (d18:1/18:1) [M - H]$^-$, *m/z* 562

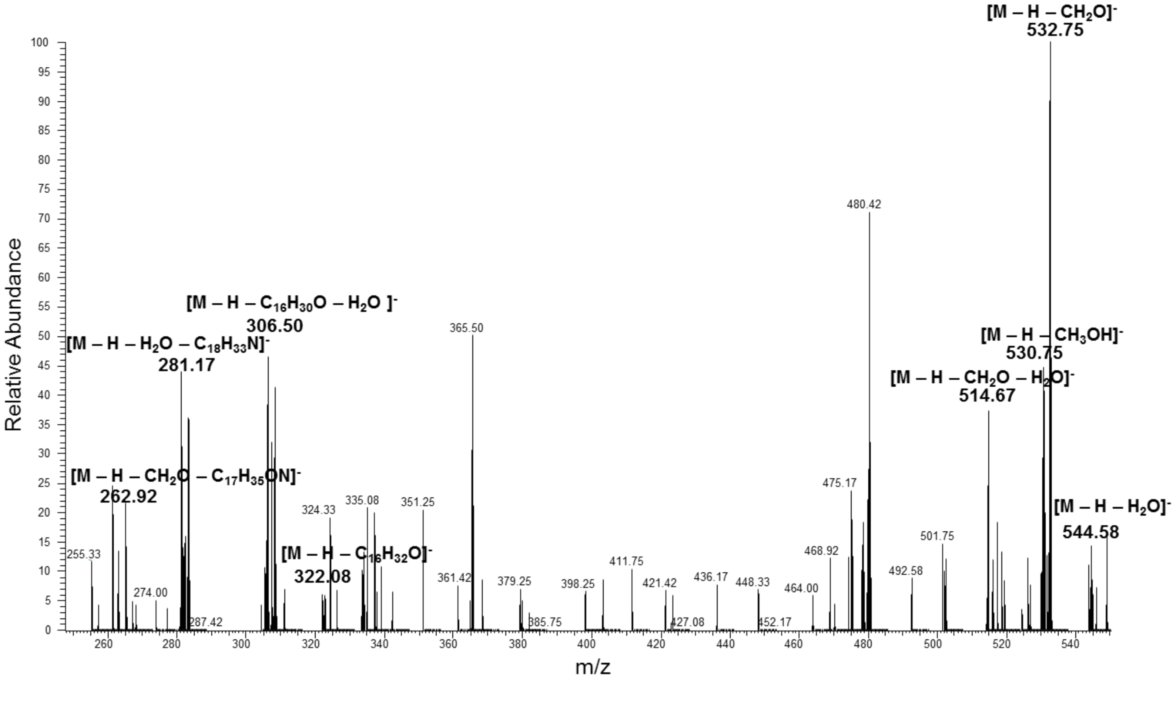

Compound #9

Cer (d18:1/18:0) [M - H]$^-$, *m/z* 564

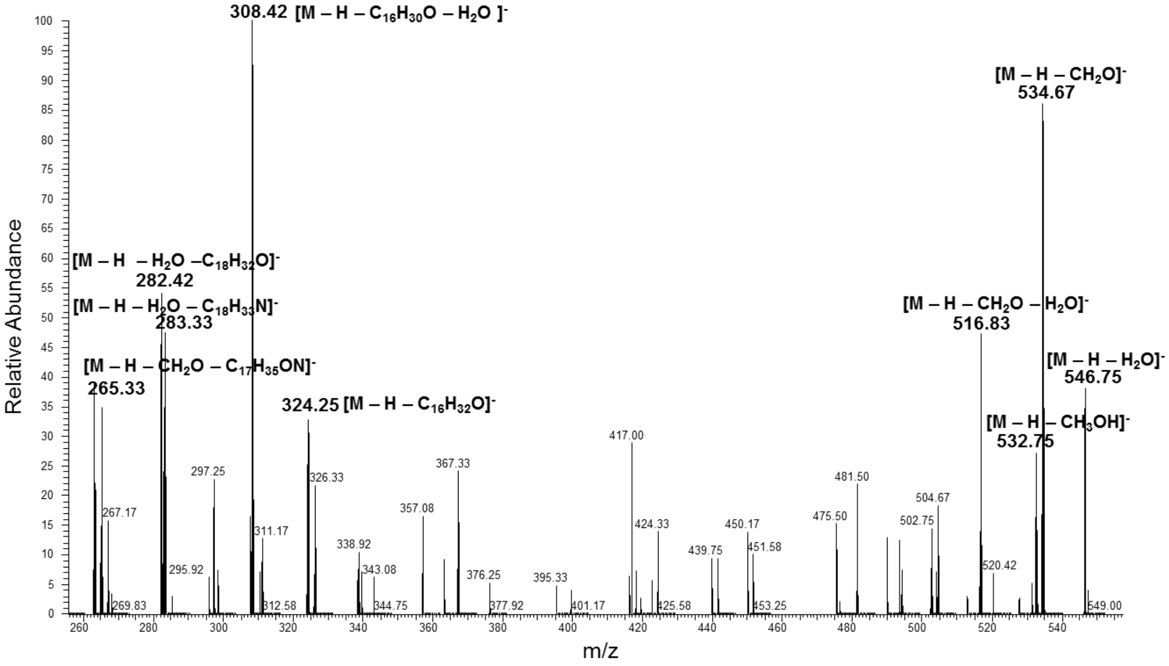

Compound #10

Cer (d18:0/18:0) [M - H]$^-$, *m/z* 566

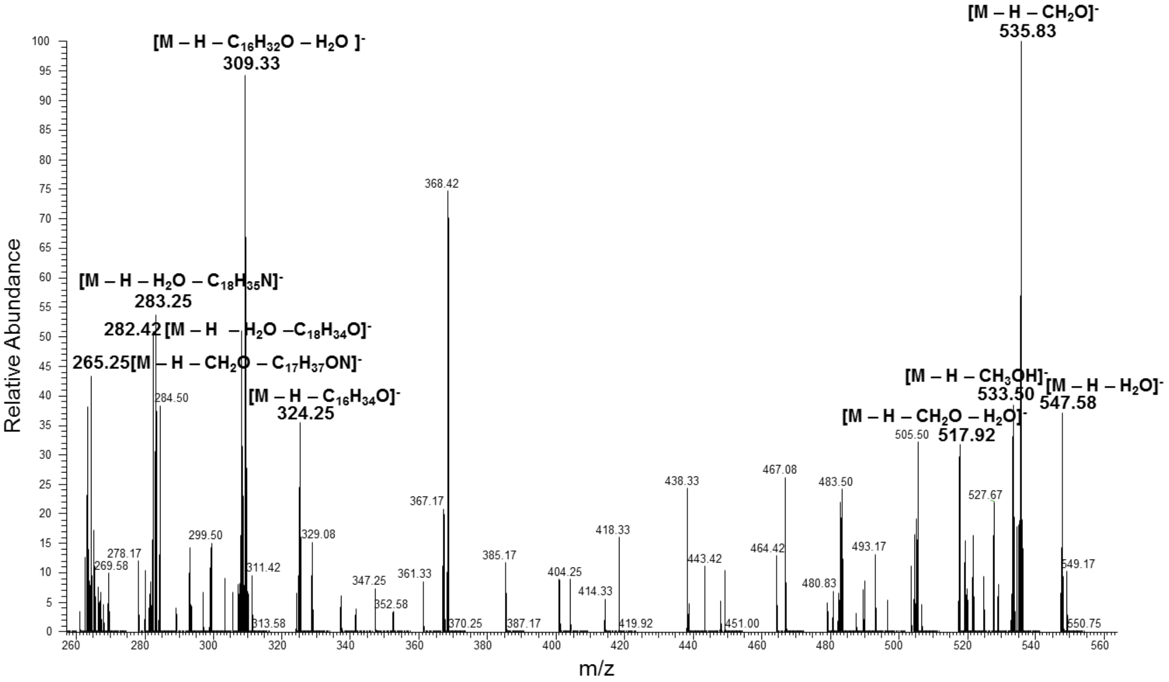

Compound #11

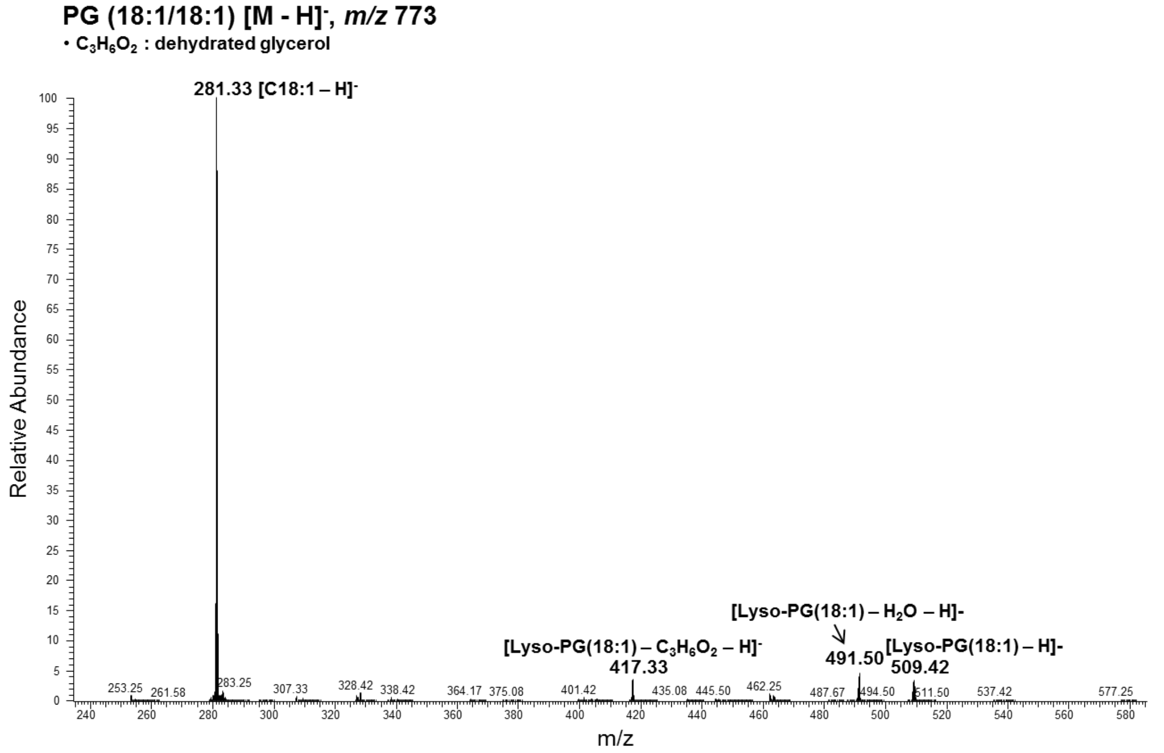

Compound #12

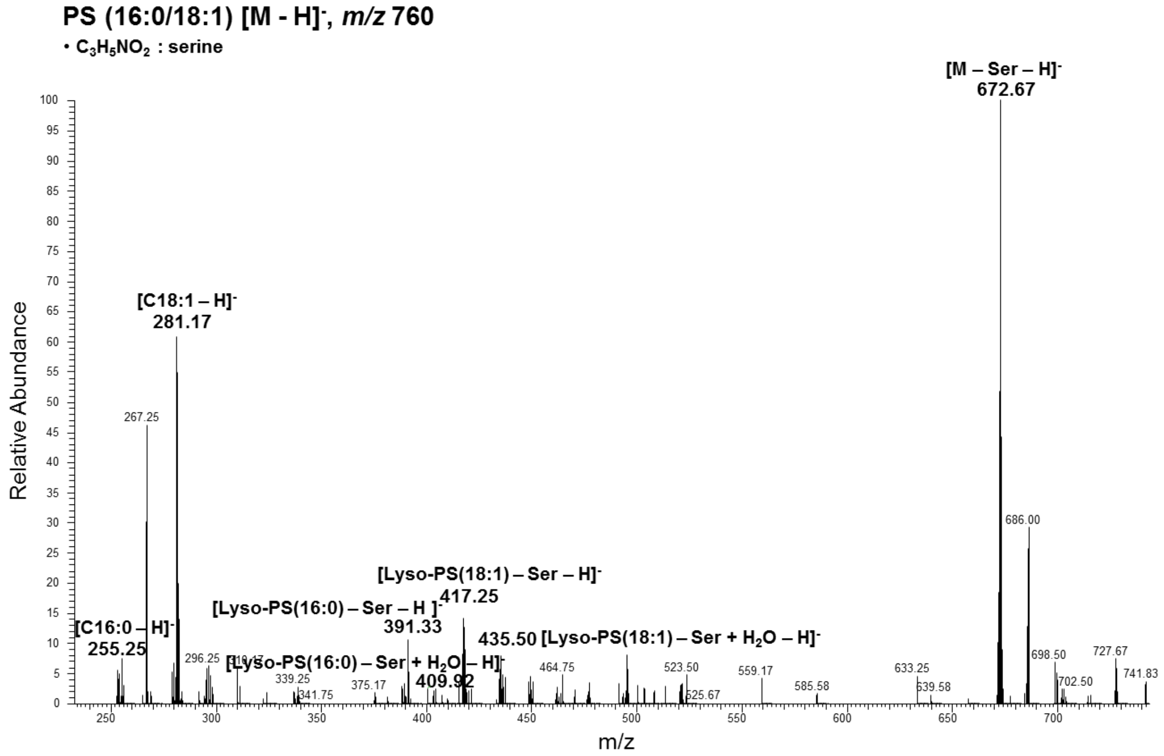

Compound #13

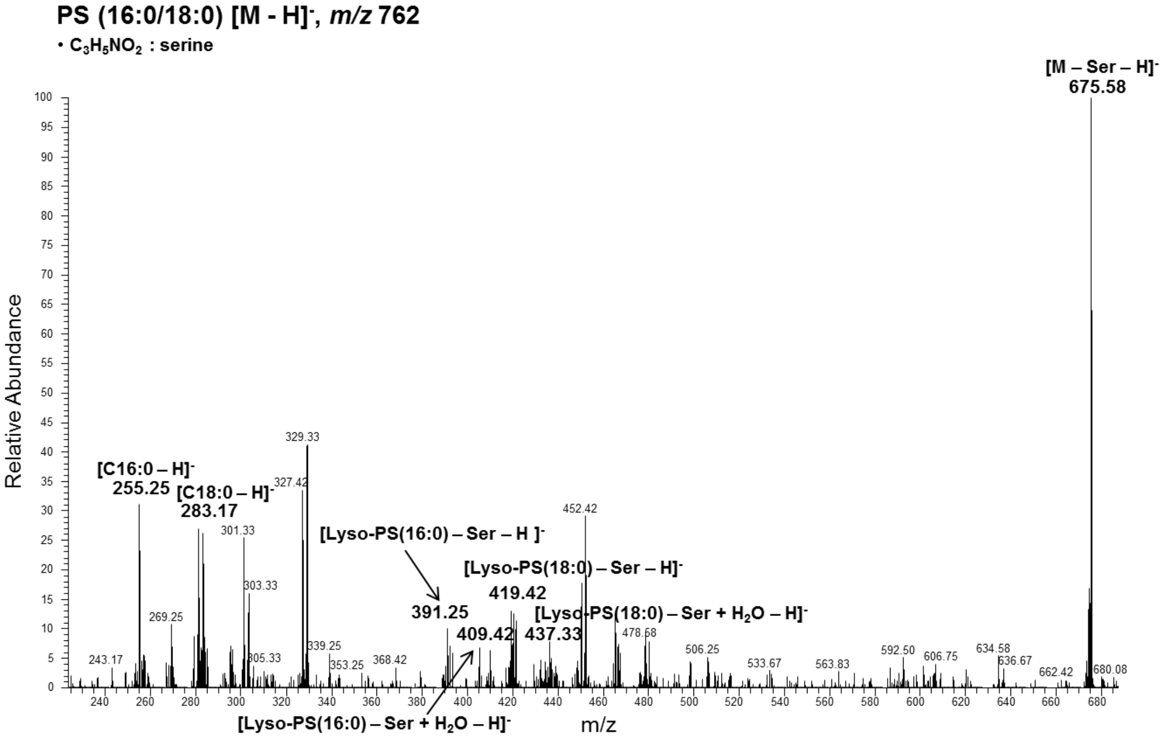

Compound #14

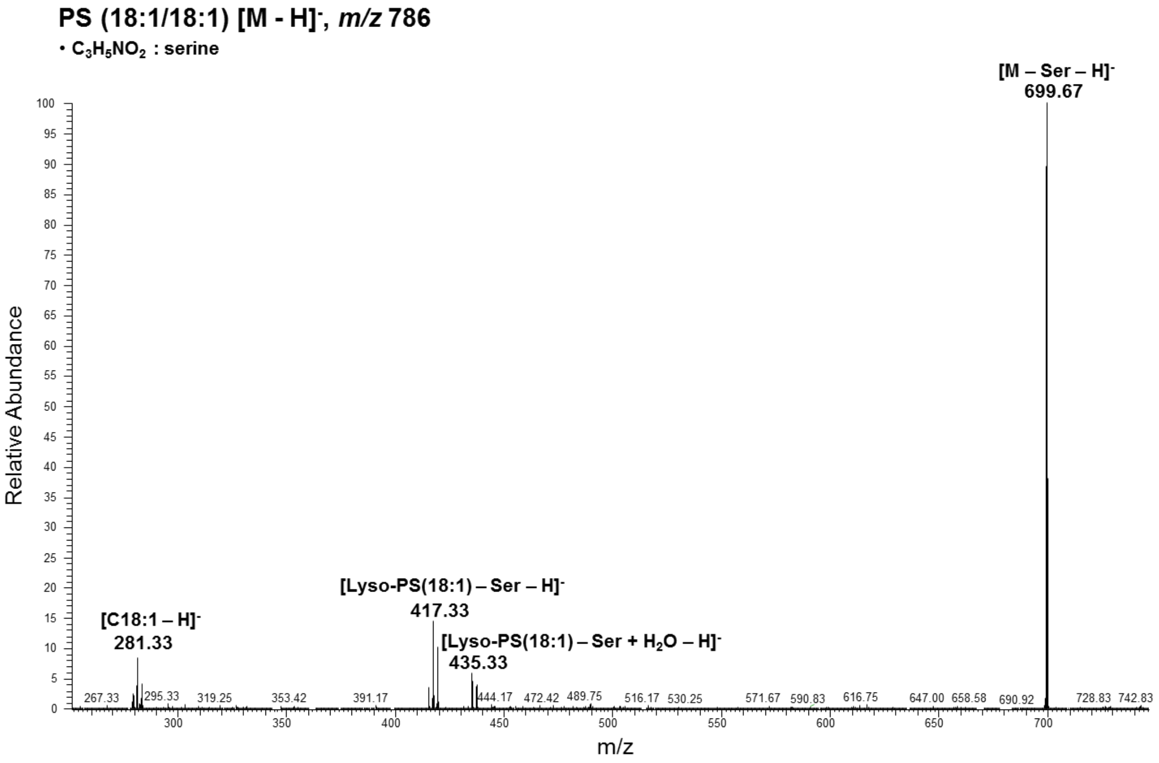

Compound #15

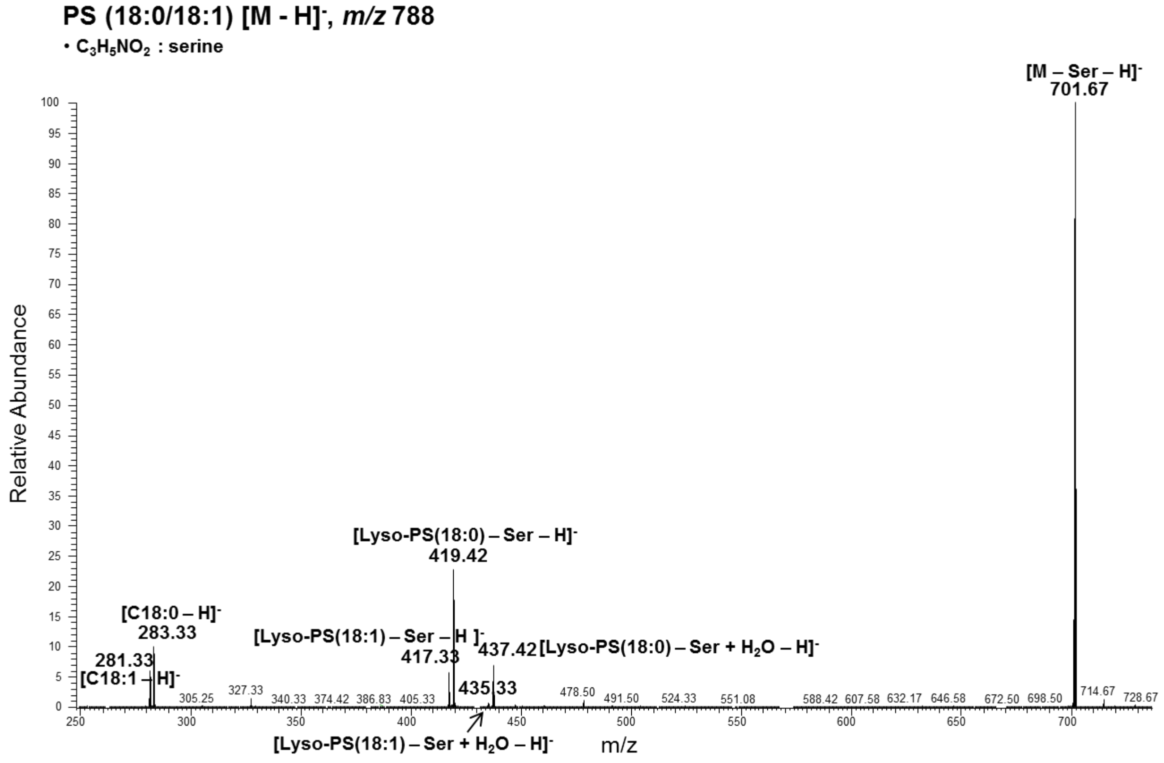

Compound #16

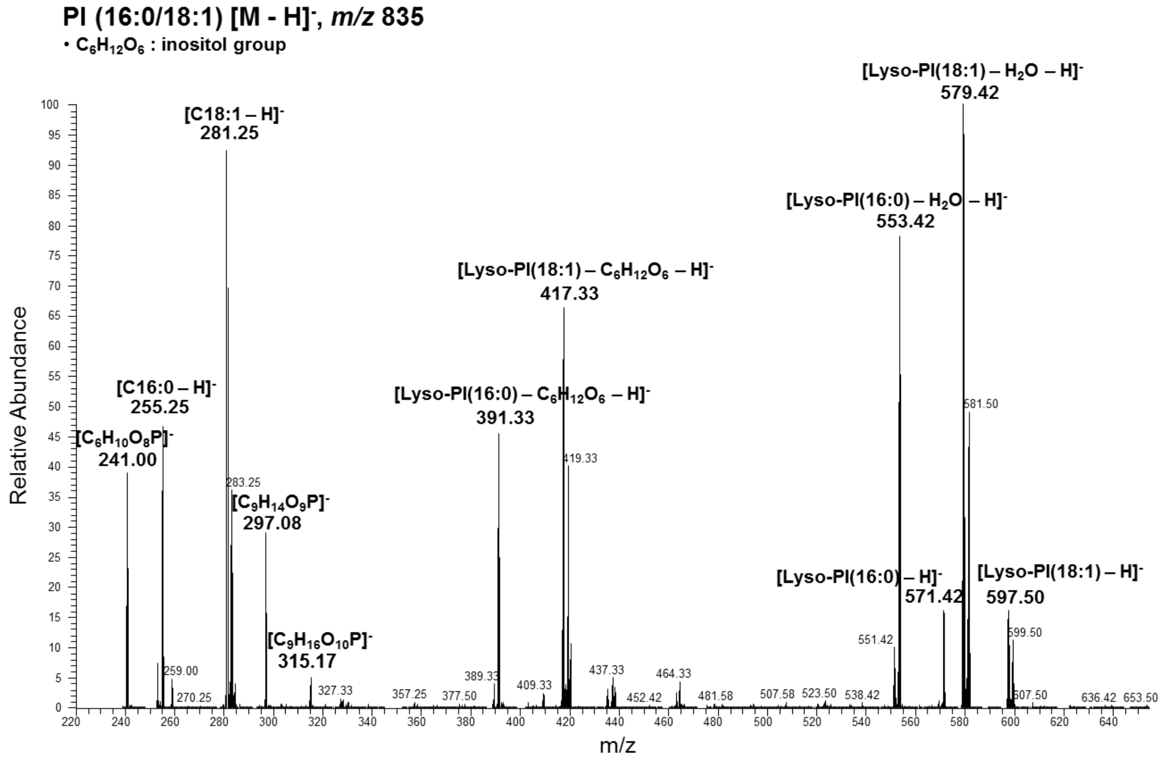

Compound #17

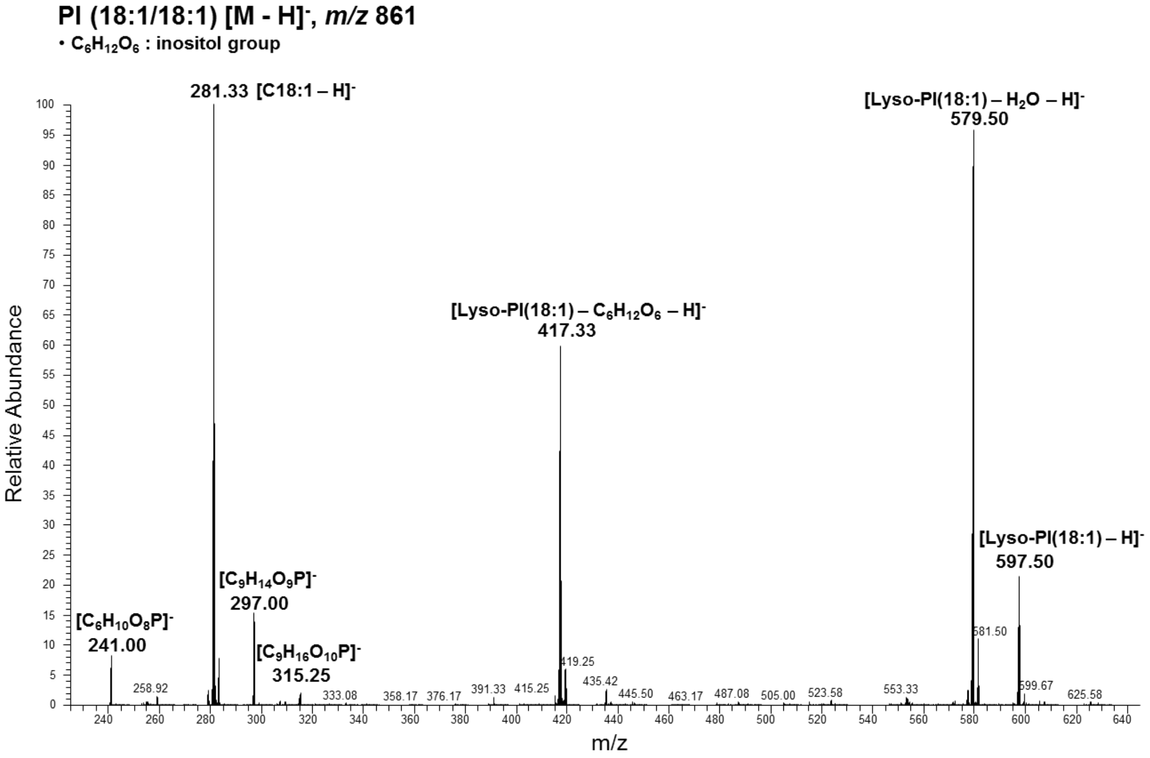

Compound #18

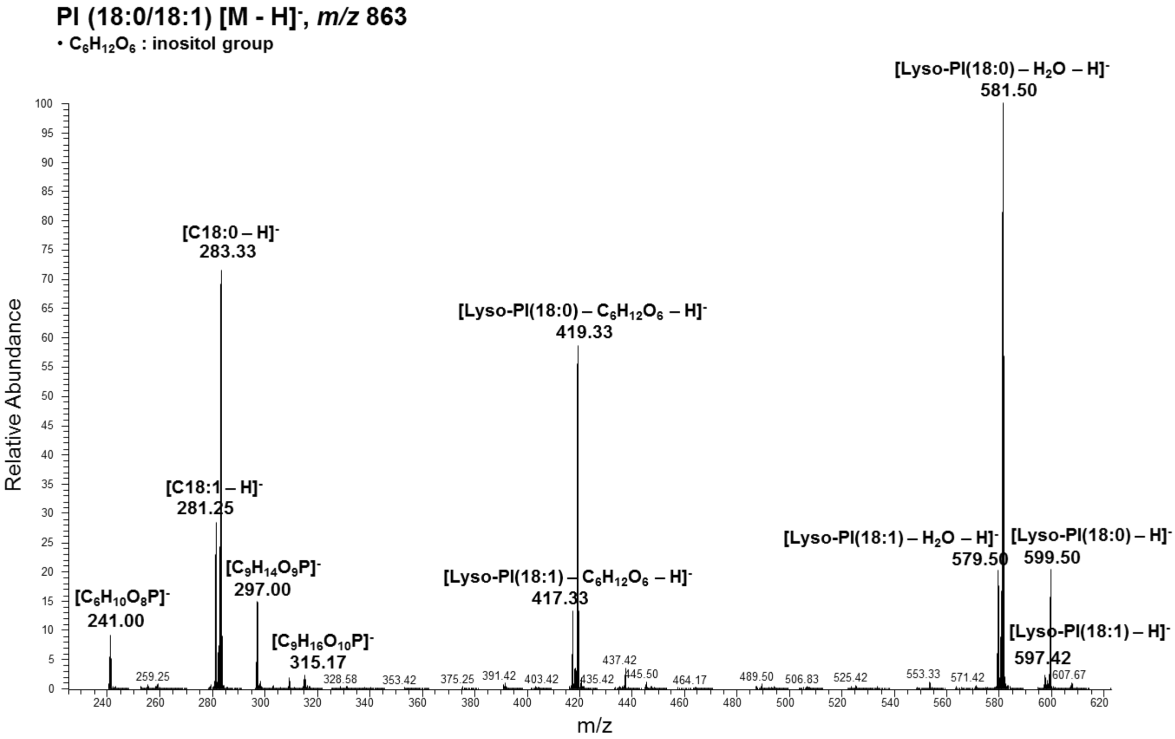

Supplementary Figure S2. Fragmentation mass spectra of the metabolites and lipids which were highly correlated with separation between COR-treated and control groups in the melanoma cell lines (A375 and A2058) based on the S-plots and VIP values from OPLS-DA analysis.

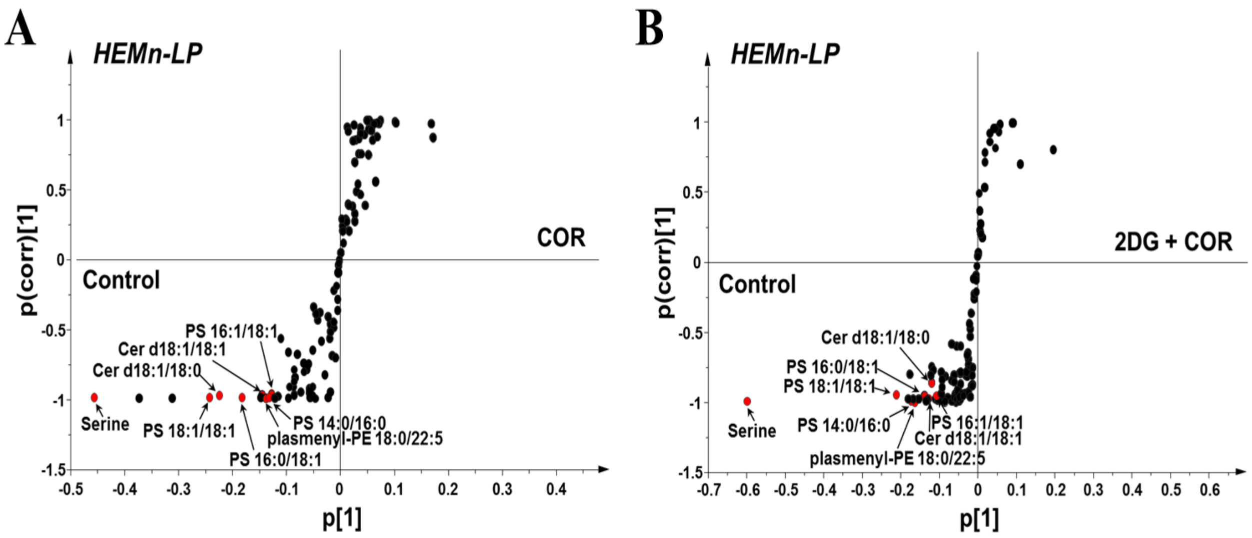

Supplementary Figure S3. The S-plots derived from metabolite and lipid profiling data of normal melanocytes (A) in the control vs. COR treatment groups and (B) in the control vs. 2-DG + COR treatment groups. The major metabolites and lipids for the discrimination between control and COR treated groups were selected based on a p(corr) cutoff value of >|0.8| and VIP value of >1.0. These compounds are highlighted using red filled circles.
